# RNA sequencing and proteomic analysis of diagnostic biopsies from colorectal and pancreatic cancer

**DOI:** 10.1016/j.bbrep.2026.102846

**Published:** 2026-11-02

**Authors:** Veronica Nordlund, Margrete Haram, Eva Hofsli, Oluf Dimitri Røe, Catharina de Lange Davies, Robin Mjelle

**Affiliations:** a Department of Physics, Norwegian University of Science and Technology, Trondheim, Norway; b Cancer Clinic, St. Olav's University Hospital, Trondheim, Norway; c Department of Radiology and Nuclear Medicine, St. Olav's University Hospital, Trondheim, Norway; d Department of Clinical and Molecular Medicine, Norwegian University of Science and Technology, Trondheim, Norway; e Department of Oncology, Levanger Hospital, Nord-Trøndelag Health Trust, Levanger, Norway; f Department of Pathology, St. Olav's University Hospital, Trondheim, Norway

**Keywords:** Colorectal cancer, Pancreatic ductal adenocarcinoma, Metastasis, Proteomics, RNA sequencing, Immune cell infiltration, Gene ontology enrichment

## Abstract

**Background:**

Colorectal cancer (CRC) and pancreatic ductal adenocarcinoma (PDAC) are two of the most frequently occurring and lethal cancers. Although numerous studies have investigated their transcriptional, molecular, and immunological landscapes, our current understanding remains incomplete. Both cancers pose major clinical challenges, and better understanding of their molecular and immunological profiles is needed to improve patient outcomes.

**Methods:**

We conducted total RNA sequencing and liquid chromatography–mass spectrometry–based proteomic profiling of 29 diagnostic biopsies from both primary tumors and metastatic sites in 16 CRC and 11 PDAC patients. Due to a lack of tissue, PDAC primary tumors were only analyzed by proteomics. Data analysis involved RNA-to–protein correlation analysis, multidimensional scaling, sample-to-sample Pearson correlations visualized as heatmaps with unsupervised clustering, differential expression analysis, gene ontology (GO) enrichment analysis, and immune cell deconvolution using CIBERSORTx.

**Results:**

Analyzing all available biopsies, RNA sequencing identified 46,492 expressed transcripts and proteomic analysis detected 6973 proteins, with 5563 genes common to both datasets. The mean absolute RNA–to-protein Spearman correlation was 0.241. In CRC, exploratory differential expression analysis identified 5 transcripts and 99 proteins differing between metastases and primary tumors. In PDAC, metastasis-to-primary tumor comparison was limited to proteomics and identified 305 differentially expressed proteins. In unsupervised clustering based on sample-to-sample correlation, CRC and PDAC biopsies formed two separate clusters. GO enrichment analysis demonstrated distinct patterns of up- and downregulation across molecular functions, cellular components, and biological processes in metastases compared with primary tumors. Inferred immune cell fractions indicated high levels of CD4 T memory resting cells and M0 macrophages.

**Conclusions:**

This exploratory study supports the feasibility of combined RNA sequencing and proteomic profiling from limited FFPE diagnostic biopsies and highlights proteomics as an important complement to transcriptomics. These findings enhance our understanding of biological and immunological heterogeneity and require further validation in larger independent cohorts for future development of prognostic markers and novel immunotherapeutic strategies.

## Highlights

• Proteomic analyses suggest reduced levels of ribosomal proteins in CRC and PDAC metastases.
• CRC metastases show enrichment in proteins associated with G-protein and GDP binding.
• PDAC metastases show enrichment in proteins linked to ADP and ATP metabolism.
• Actin-related proteins appear to be increased in PDAC metastases.

## 1 Background

Colorectal cancer (CRC) and pancreatic ductal adenocarcinoma (PDAC) are the two most frequently occurring gastrointestinal cancers [1]. PDAC is among the most lethal cancers worldwide, with a 5-year survival rate of less than 10% [1], [2]. Surgical resection remains the only curative treatment with a 5-year survival rate of only 22% [3], and the poor survival is due to a high rate of recurrence, late-stage or unresectable disease [4], [5]. First-line palliative treatment of inoperable PDAC is combination chemotherapy with FOLFIRINOX (fluorouracil, irinotecan, oxaliplatin and calcium folinate) or nab-paclitaxel-gemcitabine [6]. PDAC is highly resistant to chemotherapy [7], and the relative five-year survival for these patients is around 3% [1]. Poor outcomes highlight the need for a deeper understanding of the biology of PDAC to discover new treatment options and improve patient outcomes.

CRC is the second most common cancer worldwide [8], and around 20% of these patients present with metastases [1]. First-line treatment of metastatic CRC is chemotherapy with FOLFIRI (fluorouracil, irinotecan and calcium folinate) or FOLFOX (fluorouracil, oxaliplatin and calcium folinate) combined with either a vascular endothelial growth factor (VEGF-) or epidermal growth factor receptor (EGFR-) antibody [9]. The five-year survival of the patients presenting distant metastases at the time of diagnosis is approximately 13% [1].

Immunotherapy, particularly with immune checkpoint inhibitors (ICIs), enhances the immune system's ability to recognize and attack tumor cells [10]. The effectiveness of this treatment depends on the tumor's immune microenvironment [11]. Tumors can be classified as “hot” or “cold” based on immune cell infiltration, where hot tumors, rich in cytotoxic T cells, are more likely to respond to ICIs [12]. Cold tumors are typically able to suppress the immune response, leading to poor therapeutic outcomes [12], [13].

CRC tumors can be either hot or cold. Microsatellite instability-high (MSI-high) CRC tumors are generally considered immunologically hot [14] and respond well to ICIs, while microsatellite stable (MSS) CRC tumors do not [15], [16]. This has led to MSI testing being recommended as standard clinical practice [9]. In contrast, most PDAC tumors are immunologically cold and show resistance to immunotherapy [17], [18]. A better understanding of the immune microenvironment is necessary to discover immunotherapeutic strategies. Immune cell infiltration in tumors also has prognostic significance [19], [20], [21], with for example high densities of tissue-resident memory CD8 T cells being linked to improved survival in CRC [22] and CD4 T cells being a prognosis of improved overall survival across several cancer types [23]. Therefore, characterizing the immune cell composition of tumors is useful for prognosis, discovering new targets for immunotherapy, and guiding precision treatment strategies.

A major challenge for effective chemotherapy in solid tumors is the limited uptake of drugs [24], [25]. The major barriers to successful drug delivery are the short circulation time, inefficient extravasation across the capillary wall, restricted penetration through the extracellular matrix composed of collagen and a gel of glycosaminoglycans, and sequestration by stromal cells such as fibroblast and immune cells [24]. While these barriers exist for all solid tumors, in PDAC, which is characterized by the dense desmoplastic stroma and poor vascularization, delivery of drugs is reported to be particularly challenging [26]. Furthermore, the heterogeneity of cancer poses an additional challenge to successful treatment [27].

Recently, two clinical studies aiming to improve tumor drug uptake were published. Patients diagnosed with inoperable PDAC and metastatic CRC were treated with ultrasound and microbubbles in combination with standard chemotherapy [28], [29]. In addition to a large interpatient variation, we observed that liver metastases in the same patient responded differently to the same treatment [28]. Previous studies have highlighted tumor heterogeneity at the molecular level, including transcriptomic and proteomic differences [30]. However, the variations of the molecular and biological landscape of cancer are far from fully understood, and more characterization of both intra- and interpatient variation is needed to improve precision medicine and guide more effective patient-specific treatment decisions.

In the present study, we performed RNA sequencing and proteomics analyses of biopsies from patients enrolled in the above-mentioned clinical studies, to qualitatively characterize formalin-fixed paraffin-embedded (FFPE) diagnostic biopsies. All included biopsies except one were treatment naïve. A key strength of our study is that all biopsies were obtained from patients enrolled in the same CRC or PDAC clinical trial. Although CRC and PDAC differ biologically and clinically, both represent metastatic gastrointestinal malignancies with substantial molecular heterogeneity and limited availability of diagnostic biopsy material for comprehensive molecular characterization. Analyzing both cohorts within the same experimental and bioinformatic framework allowed us to evaluate the feasibility of combined transcriptomic and proteomic profiling from limited FFPE biopsies across two clinically challenging tumor types. Our goal was to better understand and compare RNA and protein expression in primary tumors and metastases by differential expression (DE) analysis and gene ontology (GO) analysis on transcripts and proteins that were differentially expressed in metastases compared with primary tumors. Furthermore, we sought to explore the immune landscape of these tumors using CIBERSORTx immune cell deconvolution. Due to the limited number of available samples from the two parent trials, this study is of exploratory and qualitative nature, and preliminary findings need to be validated in larger cohorts.

## 2 Methods

### 2.1 Patient biopsies

29 biopsies from patients diagnosed with inoperable PDAC and CRC with metastases to the liver were analyzed. The parent trials included 17 patients with liver metastases from CRC and 20 patients with inoperable PDAC. Material from the diagnostic biopsies was available from 16 patients from the parent CRC trial and 12 patients from the parent PDAC trial. In one case, material was available from both a PDAC primary tumor and ovarian metastasis, however, the study was not designed for paired or mixed-effects modeling. In another case, two samples were available from the same CRC primary tumor, where one was analyzed by RNA sequencing and one with proteomics. 6 samples (2 CRC primary tumor, 2 CRC liver metastasis, 2 PDAC liver metastasis) were only analyzed by RNA sequencing because proteomic analysis had already been completed at the time of acquisition, and analyzing these samples separately would have introduced batch effects. The biopsies were taken for diagnostic purposes and stored by the Norwegian biobank, Biobank1. All samples were obtained from treatment-naïve tumors, except one sample that was taken from a patient with CRC who had undergone radiation and chemotherapy according to the RAPIDO regimen [31], [32] prior to this biopsy. The patient was included in the clinical study due to recurrence. Despite not being treatment naïve, this sample was analyzed alongside the others. A treatment-naïve biopsy from this patient was not available. Therefore, analyses described in sections “Sample-to-sample comparison of overall RNA and protein expression” and “Comparisons of RNA and protein expression in metastases versus primary tumors” were conducted including and excluding this sample as a sensitivity check. Due to the sparse amount of tumor tissue in some of the biopsies, not all were eligible for both RNA sequencing and proteomics. Importantly, all biopsies of PDAC primary tumors were deemed too scarce for RNA sequencing. In total, 10 samples were analyzed through proteomics only, of which two samples were excluded after proteomic analysis. One was a CRC liver metastasis excluded due to reduced proteome coverage (2982 quantified proteins compared with a median of 5350 in retained samples). The other was a PDAC primary tumor excluded due to markedly reduced MS/MS identification rate (9.1% compared with 19-35% in other runs). No additional samples were excluded due to low RNA library complexity, low peptide identifications, or other quality-control criteria. Survival of patients was calculated as number of days between date of first diagnostic biopsy and date of death. An overview of the biopsy site and number of patient biopsies analyzed is presented in Table 1, and a schematic overview of included samples and analysis workflow is presented in Fig. 1. Further details, including mapping of sample ID to clinical characteristics, are described in the Supplementary Material S1. The use of patient biopsies was approved by the Regional Committee for Medical and Health Research Ethics Central Norway (REK Central) (REK ID 533301).

**Table 1:** Number of samples from each biopsy source analyzed by RNA sequencing and proteomics. Counts reflect total samples analyzed by each method and do not indicate whether the same samples were used for both analyses.

	RNA sequencing		Proteomics	
	Primary tumor	Metastasis	Primary tumor	Metastasis
**CRC**	12	3	10	3
**PDAC**	0	6	6	4

**Fig. 1:**
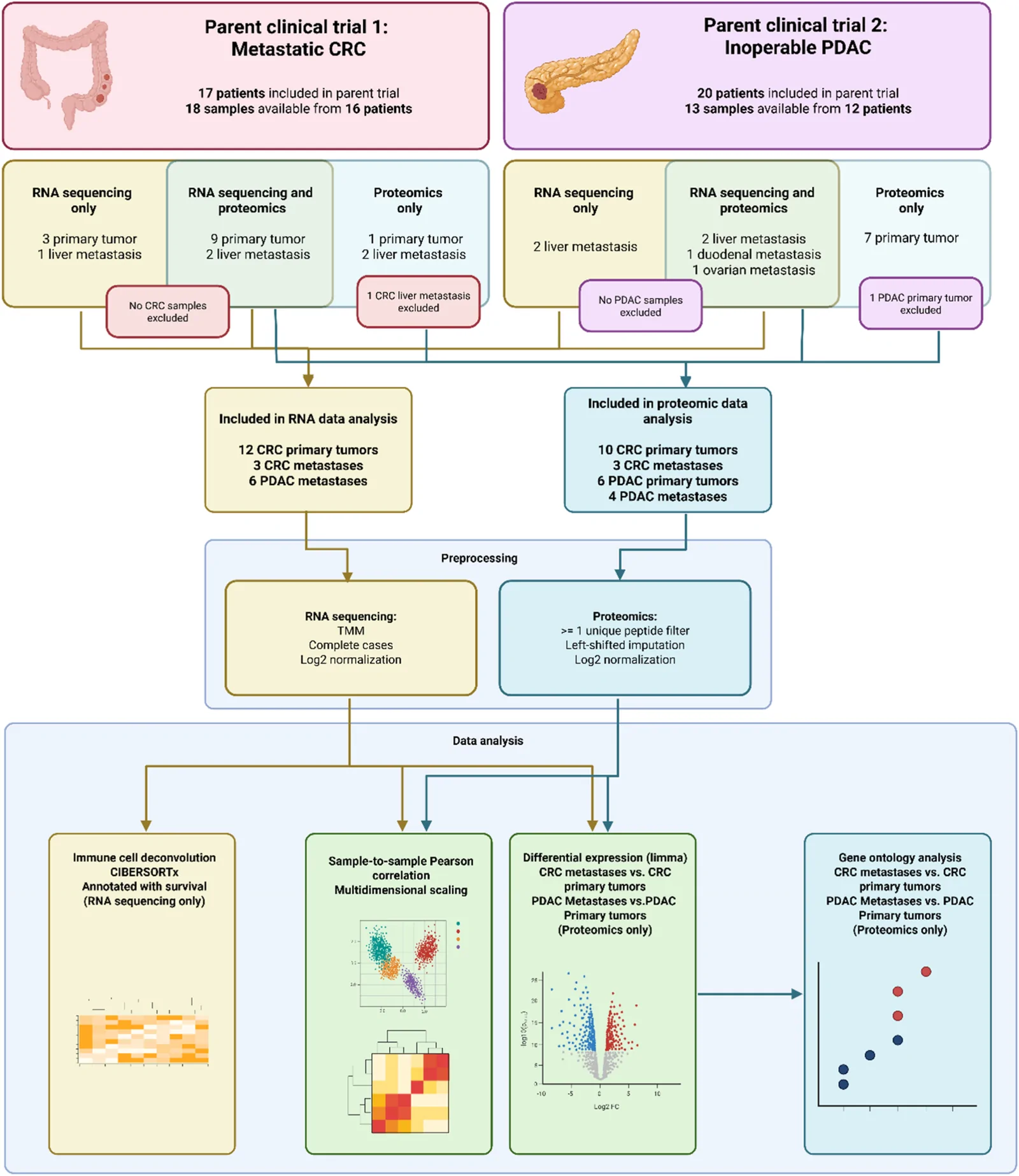
Schematic overview of patient samples included and subsequent analysis workflow. Created with BioRender.com.

### 2.2 Sample preparation and RNA sequencing and proteomics

The FFPE tumors were sectioned by the local Biobank1. Half of the sections were allocated for RNA isolation; the rest was reserved for proteomic analysis. In FFPE samples with scarce tumor material, all sections were reserved for proteomic analysis. RNA isolation was performed by Biobank1, details provided in Supplementary Material S2. The library preparation and sequencing analysis were provided in close collaboration with the Genomics Core Facility (GCF), Department of Clinical and Molecular Medicine, Norwegian University of Science and Technology (NTNU), details provided in Supplementary Material S3. RNA quality was assessed using DV200 and RIN. Across RNA-sequenced samples, the median DV200 was 47.5% (range: 0–92%), and the median RIN was 2.3 (range: 1.0–2.8). Sample-level RNA quality metrics are provided in Supplementary Material S1.

Liquid chromatography mass spectrometry (LC-MS) based identification and quantification of proteins was performed by the Proteomics and Modomics Experimental Core Facility (PROMEC), NTNU, details are provided in Supplementary Material S4. Peptides were analyzed on a timsTOF Pro mass spectrometer coupled to a nanoElute HPLC system (Bruker Daltonics) using DDA-PASEF acquisition. Raw data were processed in MaxQuant (v. 2.4.1.0) against the human UniProt reference proteome [33] with the internal contaminant database enabled. Peptide-spectrum matches, protein groups, and modification sites were filtered to a 1% false discovery rate using a target-decoy strategy. Label-free quantification was performed using the MaxLFQ algorithm with match-between-runs enabled. Protein quantification was based exclusively on unique peptides, requiring a minimum of one unique peptide for quantification.

#### 2.2.1 Public data from TCGA

Data from The Cancer Genome Atlas (TCGA) was downloaded from https://portal.gdc.cancer.gov, choosing the cancer types pancreatic adenocarcinoma (PAAD) and colon adenocarcinoma (COAD). Processed RNA sequencing and proteomic (Reverse Phase Protein Array) data was downloaded and used directly in the statistical analysis. RNA sequenced COAD data included 780 samples, of which 745 were primary tumors and 35 were metastases. Proteomic COAD data included 423 samples with no metastases. PAAD data included 113 samples of primary tumors which were all analyzed by both RNA sequencing and proteomics.

### 2.3 Data analyses

#### 2.3.1 Comparison of RNA and protein expression levels

To examine the correlation between the RNA and protein expression, UniProt protein IDs were mapped to Ensembl gene identifiers (ENSGs) using the EnsDb.Hsapiens.v86 database (Ensembl database, Bioconductor project, 2017). The gene-to-gene Spearman correlation coefficient was calculated between the log_2_ normalized matrices.

#### 2.3.2 Sample-to-sample comparison of overall RNA and protein expression

Multidimensional scaling (MDS) analysis was performed on the overall RNA or proteomic expression to examine the overall difference between biopsies, using the plotMDS function from limma [34]. Sample-to-sample Pearson correlation coefficients were calculated from the full log_2_ normalized RNA and protein expression matrices and visualized as clustered heatmaps. Hierarchical clustering of the resulting Pearson correlation matrices was performed using the default settings of ComplexHeatmap, corresponding to Euclidean distance and complete linkage.

To investigate the relationship between overall RNA or proteomic expression and clinical characteristics and outcomes, we annotated each sample with the patient's overall survival, using the date of initial diagnostic biopsy as day of diagnosis. Sample-to-sample correlations were then visualized as clustered heatmaps for each cancer type.

#### 2.3.3 Comparisons of RNA and protein expression in metastases versus primary tumors

Differential expression analysis between metastases and primary tumors was performed using the limma package, which employs empirical Bayes variance moderation [34]. For RNA sequencing data, transcripts with zero counts across all samples were removed prior to normalization. Trimmed mean of M-values (TMM) normalization was performed and expression values were transformed to log_2_-counts-per-million (CPM) using voom prior to differential expression analysis. For downstream proteomic analyses, missing LFQ values were replaced with 1 and intensities were log_2_ transformed. In the proteomic dataset, PDAC metastases were compared with PDAC primary tumors and CRC metastases with CRC primary tumors. Since there were no biopsies of PDAC primary tumors with sufficient tissue for RNA sequencing, this comparison was only done for CRC metastases versus primary tumors. P-values were adjusted using the Benjamini-Hochberg method with a false discovery rate threshold of 0.05, and proteins and transcripts were considered significantly up- or downregulated with a log_2_ fold change (FC) respectively above 0.6 or below −0.6, corresponding to a 1.5 FC cutoff, and an adjusted p-value below 0.05. Differential expression results were visualized as volcano plots, with ENSGs and UniProt IDs and differentially expressed transcripts and proteins were mapped to HUGO Gene Nomenclature Committee (HGNC) symbols using the EnsDb.Hsapiens.v86 database (Ensembl database, Bioconductor project, 2017) for readability.

#### 2.3.4 Comparisons with publicly available data

PAAD and COAD samples from TCGA were used to compare RNA and proteomic expressions as described in “Comparison of RNA and protein expression levels”. These analyses were performed to contextualize findings from the exploratory cohort and to assess whether transcript-to-protein correlation patterns were broadly consistent with observations from a larger independent dataset. Only samples with both RNA sequencing and proteomic data available were selected for this analysis. Then, sample-to-sample Pearson correlation and MDS analysis was performed on RNA sequencing data from all COAD samples using the method described in “Comparisons of RNA and protein expression in metastases versus primary tumors”. The public dataset did not include proteomic data from COAD metastases, and no RNA nor proteomic data from PAAD metastases. Thus, no comparison could be made between metastases and primary tumors for these datasets. To assess concordance between DE results obtained in the study cohort and TCGA cohort, log_2_ FCs were compared across genes present in both datasets. Spearman rank correlation analysis was performed using gene-wise log_2_ FCs derived from each DE analysis.

#### 2.3.5 Mapping of differentially expressed transcripts and proteins to GO terms

Functional enrichment analysis was performed using the enrichGO function from clusterProfiler (version 3.0.4) to discover gene ontology (GO) terms associated with the differentially expressed transcripts and proteins, focusing on molecular function, biological processes and cellular components. No experiment-specific universe was supplied to enrichGO; therefore, enrichment was performed against the default background provided by the GO annotation database. GO terms were retrieved from the Homo Sapiens organism database of Bioconductor's AnnotationHub and significant terms (adjusted p < 0.05) associated with more than 2 transcripts or proteins were visualized as dot plots. Elements in dot plots were visualized by p-value (color) and number of transcripts or proteins (size) and sorted by gene ratio, the number of transcripts or proteins associated with each term divided by the total number of input transcripts or protein.

#### 2.3.6 Immune cell deconvolution with CIBERSORTx

Immune cell fractions were inferred from RNA sequencing data using CIBERSORTx [35], [36] in absolute mode with 100 permutations. Absolute mode estimates cellular fractions as percentages of total cells, providing interpretable cell fractions without requiring a reference total cell count, which is appropriate for deconvolution of bulk samples. Quantile normalization was disabled to preserve genuine between-sample distributional differences, as per the CIBERSORTx authors' recommendations for RNA sequencing data [35], [36]. The LM22 signature matrix (547 marker genes across 22 immune cell subtypes) was used as both the reference and source profile. LM22 was originally developed and validated for microarray data but is applicable to RNA sequencing datasets. Batch correction was applied using CIBERSORTx's B-mode to correct for technical differences between the microarray-derived signature matrix and our bulk RNA sequencing mixture data. ENSGs were mapped to HGNC symbols using the EnsDb.Hsapiens.v86 database (Ensembl, Bioconductor, 2017). One hundred permutations were used to estimate statistical significance, which is sufficient for exploratory analyses while balancing computational efficiency. Proteomic data were not analyzed using CIBERSORTx because the LM22 signature matrix is transcriptome-derived and no validated proteomic immune cell signature matrix was available for these samples.

#### 2.3.7 Statistical considerations

The sample sizes in this study were determined by the available material from its parent clinical trials. Given the limited and unbalanced sample sizes, particularly for RNA sequencing as only 3 CRC metastases and no PDAC primary tumors were available, this study was not powered for definitive differential expression testing. Log_2_ FC and adjusted p-value thresholds, |log2 FC| > 0.6 and adjusted p < 0.05, were used descriptively to highlight features of interest. Because of the small group sizes, these analyses are vulnerable to both false-positive and false-negative findings, and statistical significance should therefore not be interpreted as definitive evidence of biological regulation.

## 3 Results

### 3.1 Comparison of RNA and protein expression levels

Analyzing all available biopsies, the RNA sequencing identified 46492 expressed transcripts, while the proteomic analysis detected 6973 proteins. After mapping ENSGs and UniProt IDs to unique HGNC symbols, we identified 5563 genes that were common to both datasets. Expression matrices were constructed for these genes across 15 samples for which both RNA sequencing and proteomic data were available.

The gene-to-gene Spearman correlation between mapped transcripts and proteins showed a Gaussian distribution, indicating modest concordance between RNA and protein expression (Supplementary Material S5A). The mean absolute Spearman correlation was 0.241 (standard deviation = 0.171, median = 0.214, min = 0.000, max = 0.903). The 40 genes with strongest correlation between RNA and protein expression were investigated in more detail (Supplementary Material S5B). Those with the strongest positive correlations were associated with mitochondrial function (*TOMM20*, *MRPS9*, *MRPS10*), protein degradation and the ubiquitin pathway (*USP10*, *PSMD12*, *BABAM1*, *DCAF7*), extracellular matrix and tissue structure (*BGN*, *ELN*), immune responses *(IGHV3-66*, *CRP*, *EPCAM*), and RNA metabolism and translation (*RPP25*, *MRPS9*, *MRPS10*, *SNF8*) [37], [38]. Those with the strongest negative correlations were more diverse and associated with transcription and gene regulation (*EIF5A*, *SF3B1*, *PURB*, *POU5F1*, *TCF25*), immune response (*MX2*, *SRGN*, *MNDA*, *ATRN*), microtubule dynamics regulation of the cytoskeleton (*CLASP2*, *SH3PXD2B*), cell signaling (*GNB1*), apoptosis (*PPP1R13L*, *TRIM39*, *BDH2*), protein synthesis (*RPL13A*, *EIF5A*), blood clotting (*SERPINC1*), and neuromodulation (*PTGDS*) [37], [38].

Next, we investigated the relationship between RNA-to-protein correlation and RNA and protein abundance (Supplementary Material S5C). When grouping the data in 5 equally large groups based on their RNA or protein expression level, we found no obvious increase of the mean absolute Spearman correlation and the abundance of the transcript or protein, indicating that RNA-to-protein correlations were independent of their expression level.

TCGA data contained 59427 transcripts and 487 proteins for both the COAD and PAAD datasets. Both RNA and proteomic datasets were annotated with HGNC symbols, of which 168 genes were present in both RNA and proteomic data. Consistent with our dataset, the mean transcript-to-protein correlation in the TCGA datasets was 0.246 (standard deviation = 0.203, median = 0.235, min = 0.004, max = 0.712). The distribution of gene-to-gene Spearman correlation between mapped transcripts and proteins was not Gaussian (Supplementary Material S6A), which indicates that the correlation depended on the expression level. This was confirmed by grouping data in 5 equally large groups based on their RNA or protein expression level (Supplementary Material S6C), where the absolute Spearman correlation was largest for the highest-abundance transcripts and proteins, possibly reflecting bias introduced by the panel-based proteomic assay used for the TCGA dataset. The 40 genes with the highest absolute correlation did not intersect with the highest-correlating genes in our analysis (Supplementary Material S6B). Of the 487 proteins in the TCGA proteomic datasets, only 97 overlapped with our proteomic dataset. This limited overlap likely reflects the methodological differences between our discovery-based LC-MS proteomic analysis and the TCGA reverse phase protein arrays (RPPA) platform, which is an antibody-based targeted approach with limited protein coverage and dynamic range. Given the small intersection of proteins, direct comparisons between the TCGA and our proteomic dataset were deemed unreliable.

### 3.2 Sample-to-sample comparison of overall RNA and protein expression

To examine the overall RNA and protein expression of the two cancer types, we performed unsupervised clustering with sample-to-sample comparison. Mapping of IDs to clinical characteristics is provided in Supplementary Material S1. Both for RNA and proteomics, we observed two main clusters, one with CRC biopsies and one with PDAC biopsies, indicating that the two cancer types have distinct RNA and protein expressions (Fig. 2A and B). The RNA sequencing showed that two CRC liver metastases clustered together with the CRC primary tumors (Fig. 2A). Interestingly, one CRC liver metastasis sample did not cluster with any other samples, neither CRC nor PDAC (sample 7_mCRC, Fig. 2A), indicating a distinctly different total RNA composition. This sample is from the CRC patient who had received radiotherapy and chemotherapy previously. The analysis was rerun excluding this sample (Supplementary Material S7), however, this did not change the overall clustering of the samples. The PDAC liver and duodenal metastases clustered separately from all CRC samples, while the PDAC ovarian metastasis clusters with CRC primary tumors (Fig. 2A). The Pearson correlation coefficient is larger than 0.9 for all sample-to-sample comparisons of RNA expression, demonstrating overlap in the total RNA composition regardless of metastasis site and primary tumor type.

**Fig. 2:**
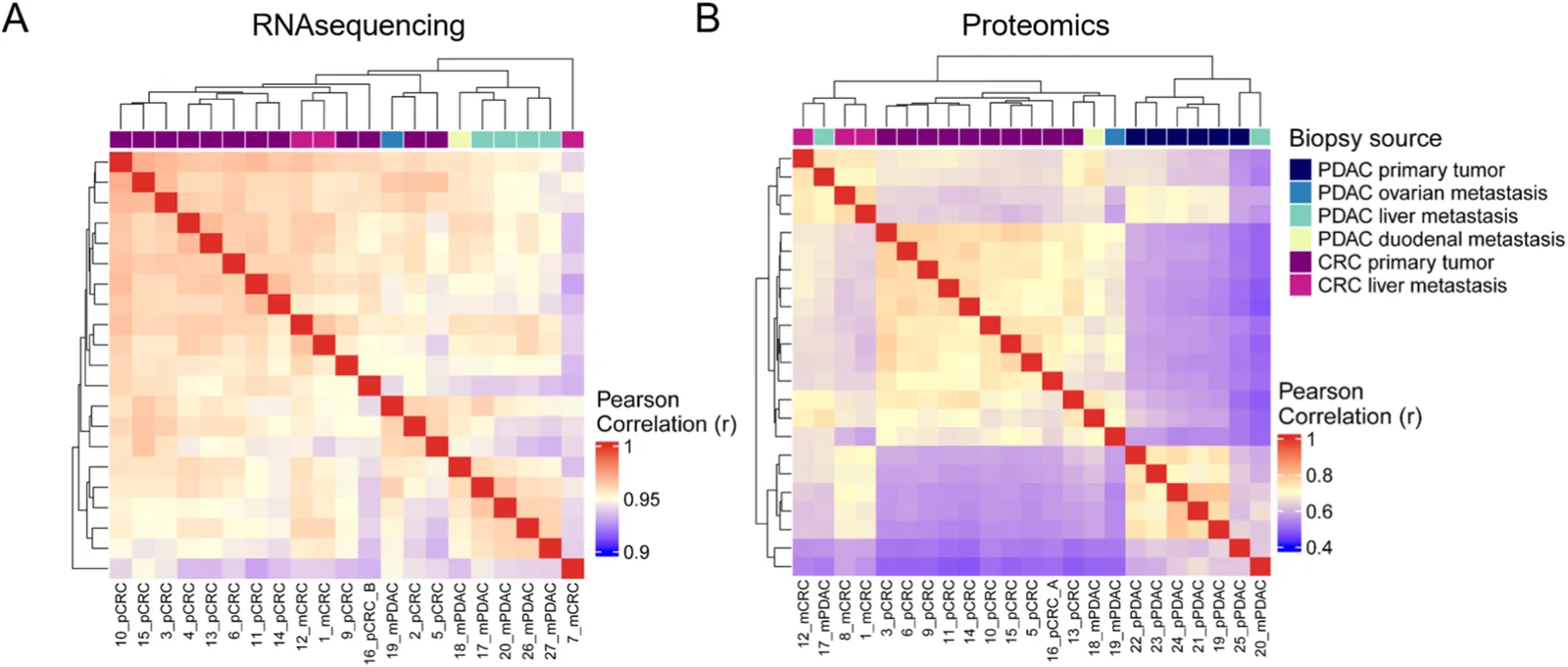
Sample-to-sample correlation heatmaps of overall protein and RNA expression. A) Overall expression of RNA in biopsies shown as a heatmap of the Pearson correlation coefficient (r) in sample-to-sample comparisons of RNA-sequenced biopsies: CRC primary tumors (n = 12), CRC metastases (n = 3), and PDAC metastases (n = 6). No PDAC primary tumors were available for RNA sequencing. Sample 7_mCRC is from a previously treated patient. B) Overall protein expression in biopsies shown as a heatmap of the Pearson correlation coefficient (r) in sample-to-sample comparisons of proteomic data: CRC primary tumors (n = 10), CRC metastases (n = 3), PDAC primary tumors (n = 6), and PDAC metastases (n = 4). Hierarchical clustering was performed on the Pearson correlation matrices using Euclidean distance and complete linkage.

In the heatmap of total protein expression, the differences between the samples were more apparent. We observed two main clusters, one containing all PDAC primary tumors and one containing all CRC biopsies, with the CRC cluster clearly separating into two sub-clusters of metastases and primary tumors (Fig. 2B). In one case, two samples from the same patient were analyzed (samples 19_mPDAC and 19_pPDAC, Fig. 2B). Interestingly, the ovarian metastasis clustered with CRC primary tumors, whereas the corresponding primary tumor was more strongly correlated with the other PDAC primary tumors. This indicates a significant intra-patient divergence between the metastasis and its matched primary tumor. Notably, PDAC metastases did not consistently cluster together, instead, the PDAC duodenal and ovarian metastases clustered with the CRC primary tumors, one PDAC liver metastasis clustered with the CRC liver metastases, and another PDAC liver metastasis clustered with the PDAC primary tumor biopsies (Fig. 2B). This pattern suggests that while the primary cancer type remains the main source of variation, the metastasis site had a greater impact on tumor protein expression than on RNA expression. The Pearson correlation coefficient was also notably lower in many sample-to-sample comparisons of protein expressions than in RNA-based comparisons. Overall, the overall RNA expression varied less between biopsy sources than total protein expression, although differences by cancer type and biopsy site were evident in both datasets. High correlations in the RNA sequencing data indicate substantial overlap in global transcriptomic composition between samples, which is expected, since correlations were calculated from the full log_2_-transformed matrices.

MDS was applied to RNA and protein expression data to assess overall variation among the samples (Fig. 3). In the RNA sequencing data, two primary clusters were observed, corresponding CRC biopsies and PDAC metastasis biopsies. The first MDS dimension captured 19% of the total variance in the RNA expression, likely reflecting overlap in transcriptomic composition between samples, as also indicated by the Pearson sample-to-sample correlation heatmap (Fig. 2A). The second dimension captured 15% of the variance, however, no obvious clustering was seen along this axis, suggesting the biopsy source was not the main contributor to the variability in the RNA expression. The proteomic data showed a stronger association between sample clustering and biopsy source. CRC and PDAC primary tumors separated along the first dimension, which captured 28% of the total variance. The second dimension captured 10% of the variability, solidifying the notion that protein expression variability was more strongly influenced by biopsy source compared with RNA expression.

**Fig. 3:**
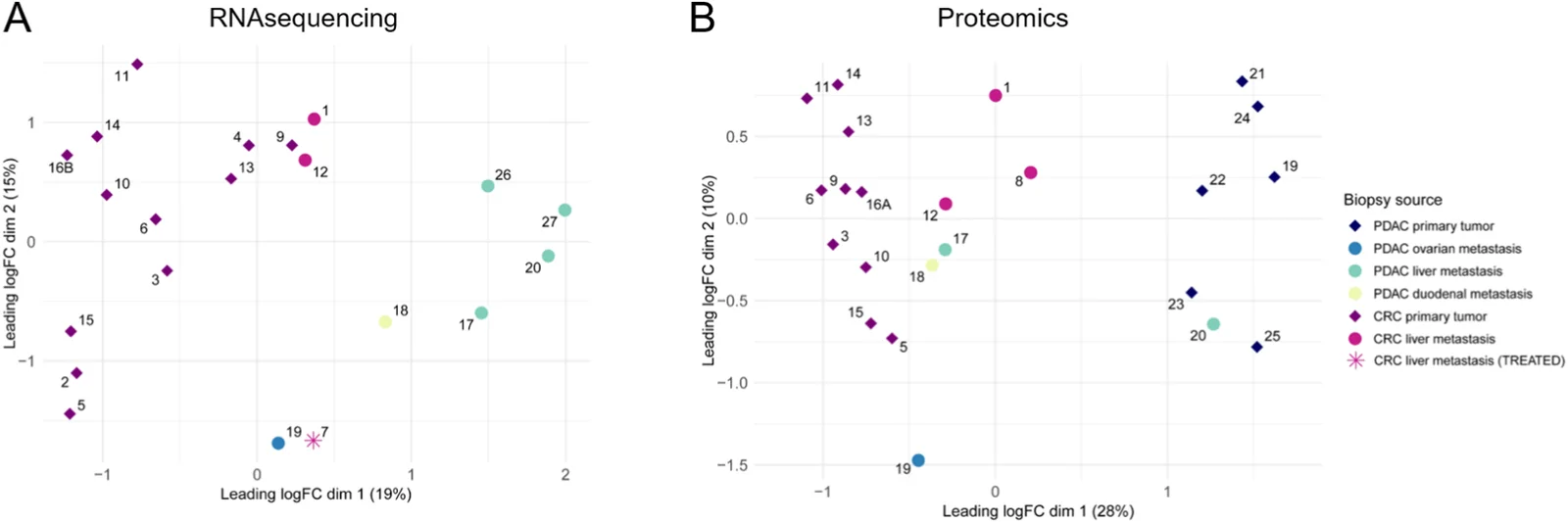
Two-dimensional (2D) multidimensional scaling (MDS) projections of overall RNA and protein expression. A) 2D MDS projection of overall RNA expression from RNA sequenced biopsies: CRC primary tumors (n = 12), CRC metastases (n = 3), and PDAC metastases (n = 6). Sample 7 is from a previously treated patient. B) 2D MDS projection of overall protein expression of CRC primary tumors (n = 10), CRC metastases (n = 3), PDAC primary tumors (n = 6), and PDAC metastases (n = 4). Dim 1 = dimension 1, dim 2 = dimension 2.

When comparing RNA and protein expression to clinical outcomes by annotating heatmaps of the sample-to-sample Pearson correlation coefficient with biopsy source and overall survival (Supplementary Material S8), no associations were identified. In PDAC biopsies, both RNA and protein expression formed two primary clusters, but these did not show a clear relationship with overall survival. In CRC biopsies, proteomic clustering revealed three clusters. One cluster contained only primary tumors, including patients with both short survival below one year and longer, indicating no consistent prognostic pattern. RNA-based clustering of CRC biopsies was less distinct and did not suggest any association with survival. One sample did not cluster with any of the other samples; this was the sample from the patient who underwent radiation and chemotherapy prior to being included in the trial. MDS analysis excluding this sample was performed, but did not change the overall clustering of the samples in the projection (Supplementary Material S9).

MDS analysis was also performed on transcriptomic data from TCGA (Supplementary Material S10), revealing two main clusters. All metastases grouped within one cluster, which also included many primary tumor samples, indicating overlap between the groups.

### 3.3 Comparisons of RNA and protein expression in metastases versus primary tumors

Based on the clusters of primary tumors and metastases (Fig. 2), a differential expression analysis was performed, comparing metastases versus primary tumors (Fig. 3, Fig. 4 and Supplementary Material S11).

**Fig. 4:**
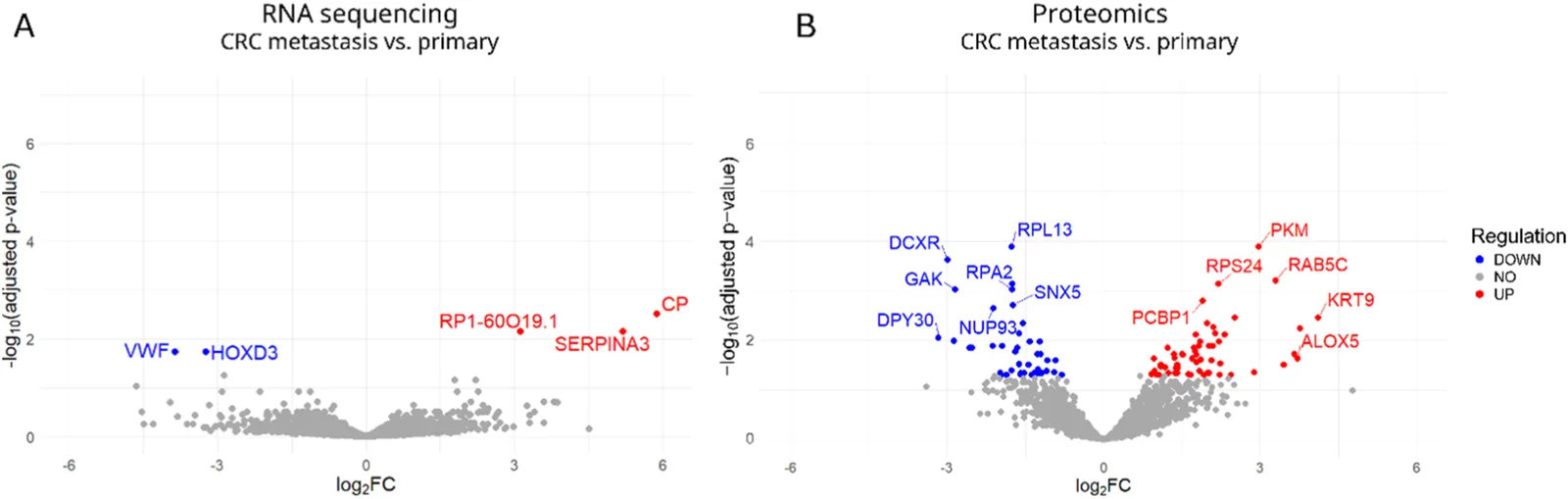
Differential expression analysis of CRC metastases compared with CRC primary tumors. A) Volcano plot showing differentially expressed transcripts in CRC metastases (n = 3) relative to CRC primary tumors (n = 12), identified by RNA sequencing. B) Volcano plot showing differentially expressed proteins in CRC metastases (n = 3) relative to CRC primary tumors (n = 10), identified by proteomics. Differential expression analysis was performed using limma. P-values were adjusted using the Benjamini-Hochberg method, and transcripts or proteins were considered differentially expressed using thresholds of |log2FC| > 0.6 and adjusted p < 0.05. Blue and red dots represent downregulated and upregulated transcripts or proteins in metastases, respectively.

When comparing the RNA expression of the CRC biopsies, five transcripts were differentially expressed between metastases and primary tumors (Fig. 4A). *VWF* and *HOXD3* were downregulated, whereas *RP1-60O19.1*, *CP*, and *SERPINA3* were upregulated. Analyzing the RNA CRC samples with 7_mCRC excluded also yielded 5 differentially expressed transcripts (Supplementary Material S11), where *VWF*, *TSPAN11*, and *CXCL14* were downregulated, and *RP1*-*60O19.1* and *CP* were upregulated. Thus, three transcripts (*VWF, RP1-60O19.1,* and *CP*) were identified as differentially expressed both including and excluding sample 7_mCRC, while two transcripts (*TSPAN11* and *CXCL14*) were unique to the analysis excluding sample 7_mCRC. The protein expression analysis showed 99 differentially expressed proteins, 42 downregulated and 57 upregulated (Fig. 4B).

In the comparison of protein composition of PDAC metastases versus primary tumors, 151 proteins were downregulated and 154 upregulated in the metastases (Fig. 5). When mapped to gene names for comparison, none of the differentially expressed transcripts found in the RNA comparison corresponded to differentially expressed in the protein comparison. However, two proteins were downregulated in both PDAC and CRC metastases, the large ribosomal subunit protein uL16 and the replication protein A 32 kDa subunit, coded for by the *RPL10* and *RPA2* gene, respectively [39].

**Fig. 5:**
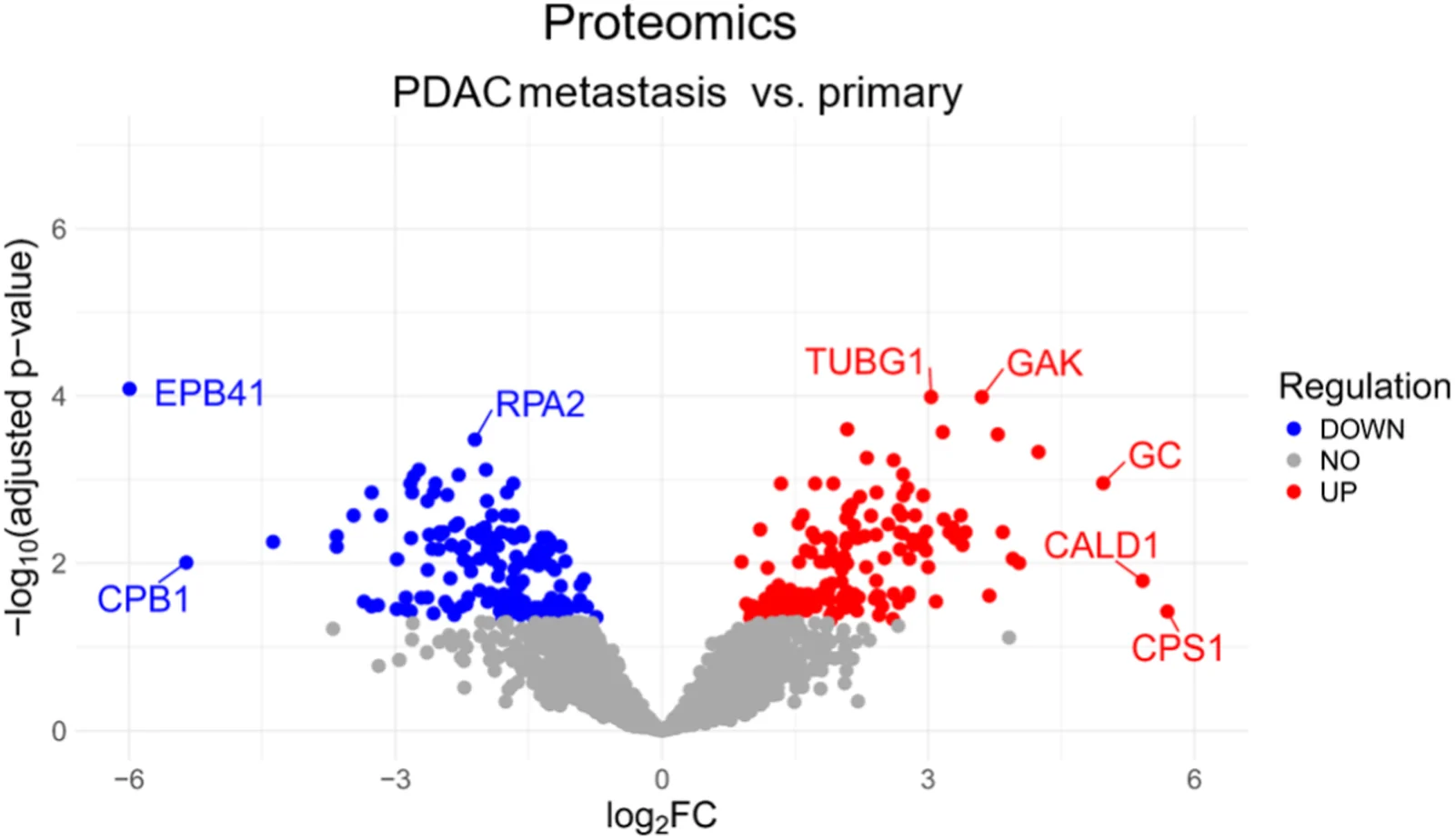
Differential expression analysis of PDAC metastases (n = 4) compared with primary tumors (n = 6). Volcano plot showing differentially expressed proteins (the most differentially expressed mapped to gene names) in metastases relative to primary tumors, identified by proteomics. The blue and red dots represent proteins that are respectively downregulated or upregulated in metastases compared with the primary tumors. Differential expression analysis was performed using limma. P-values were adjusted using the Benjamini-Hochberg method, and proteins were considered differentially expressed using thresholds of |log2FC| > 0.6 and adjusted p < 0.05. Blue and red dots represent downregulated and upregulated proteins in metastases, respectively.

In the comparison of TCGA COAD metastases versus primary tumors (Supplementary Material S12, S13), 969 upregulated and 844 downregulated transcripts were identified. Although no overlapping significantly differentially expressed genes were identified between TCGA and our cohort, genome-wide log fold changes demonstrated moderate positive concordance between the cohorts (Spearman's ρ = 0.35, p < 2.2 × 10^−16^).

### 3.4 Mapping of differentially expressed transcripts and proteins to GO terms

Next, we investigated if the differentially expressed transcripts and proteins (|log2 FC| > 0.6 and adjusted p < 0.05) in metastases versus primary tumors were associated with specific GO terms, focusing on biological processes, molecular functions, and cellular components. Because the RNA-based metastasis-to-primary tumor comparison of CRC samples identified only five differentially expressed transcripts, RNA-based GO enrichment results were considered underpowered and were not presented in the Results section. To reduce overinterpretation of GO terms supported by very few features, only terms supported by at least three differentially expressed proteins or transcripts were included in the figures in the Results section. Under this criterion, the Results section only contains GO terms from proteomic enrichment results. The complete GO enrichment output, including RNA-based results and terms not shown in the main figures, is provided in Supplementary Material S14.

GO enrichment analysis of molecular functions identified four upregulated proteins in the CRC metastases associated with G protein activity and GDP binding (Fig. 6A), while the downregulated proteins were associated with phosphatidylinositol binding and ribosome structure (Fig. 6B), suggesting altered involvement of signaling pathways in metastases compared with primary tumors.

**Fig. 6:**
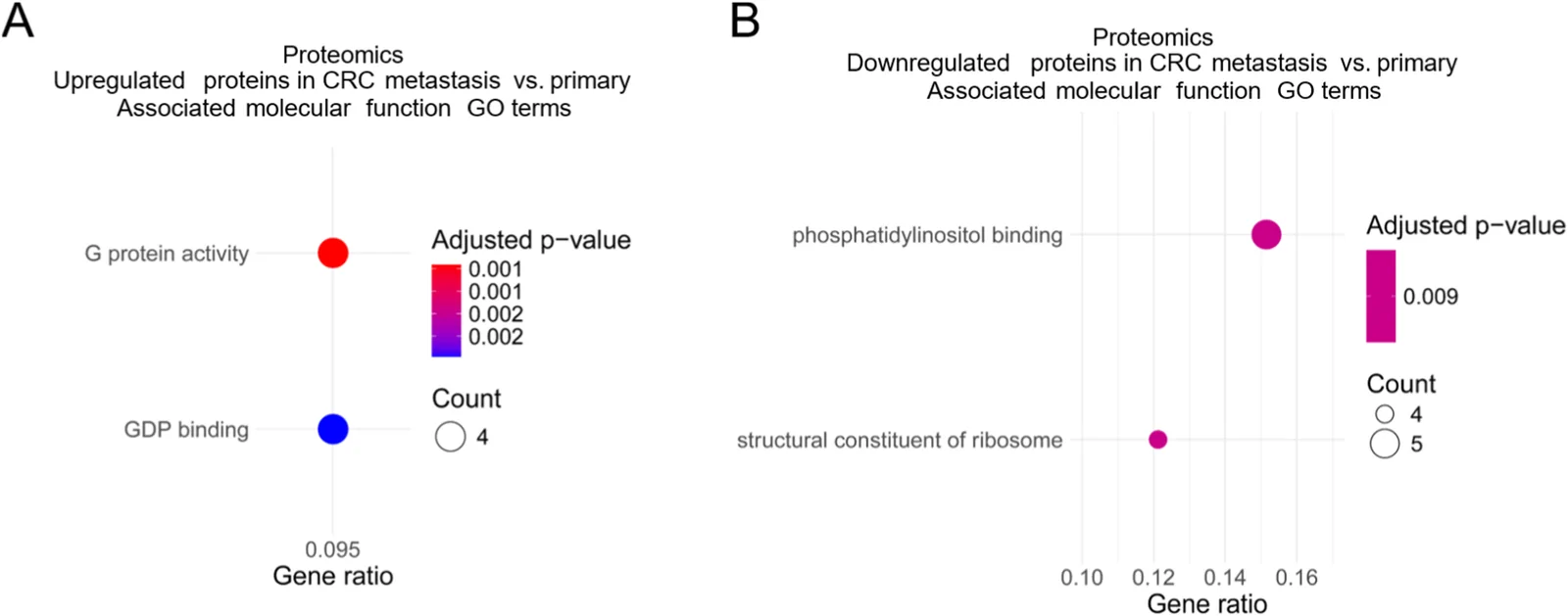
Molecular function gene ontology (GO) terms associated with differentially expressed proteins in CRC metastases (n = 3) relative to primary tumors (n = 10). A) GO terms associated with proteins upregulated in CRC metastases. B) GO terms associated with proteins downregulated in CRC metastases. GO enrichment analysis was performed using clusterProfiler enrichGO. Only GO terms supported by at least three differentially expressed proteins are shown. Dot size indicates the number of contributing proteins, and dot color indicates the adjusted p-value.

GO analysis of cellular components revealed substantial differences between metastases and primary tumors (Fig. 7). Upregulated proteins in the metastases were largely related to granules, and blood microparticle (Fig. 7A). Blood microparticle was also seen in GO enrichment analysis of RNA sequencing data (Supplementary Material S14). Consistent with the observed molecular functions, several downregulated proteins in the CRC metastases were associated with the ribosome (Fig. 7B), which may suggest reduced ribosomal activity in CRC metastases relative to primary tumors.

**Fig. 7:**
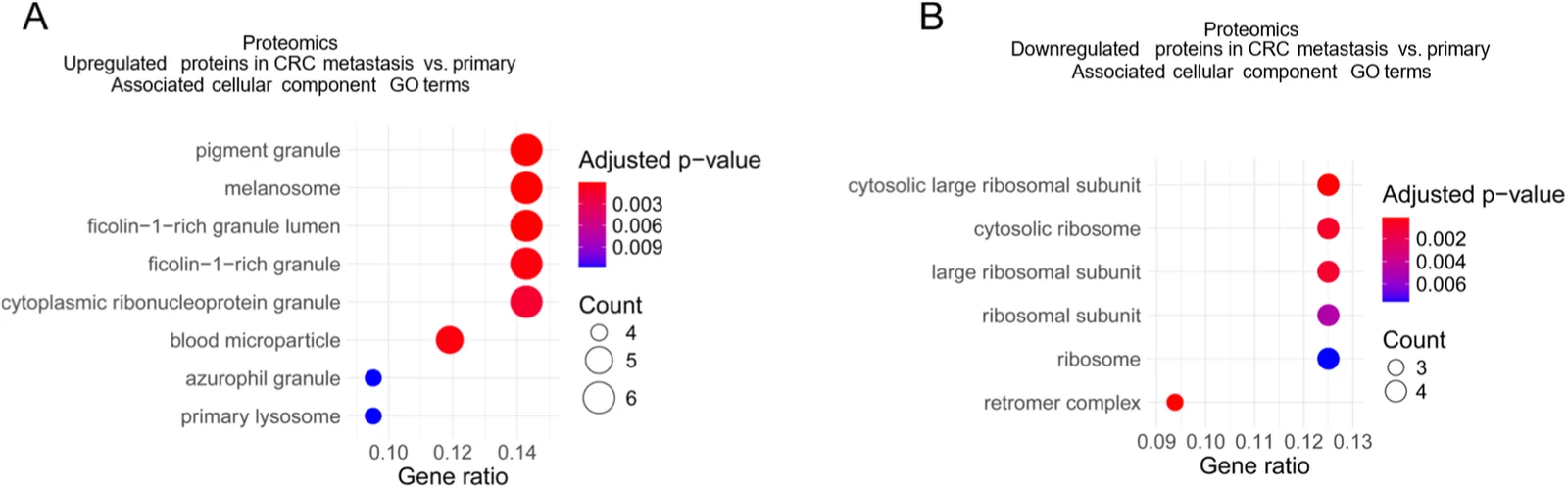
Cellular component gene ontology (GO) terms associated with differentially expressed proteins in CRC metastases (n = 3) relative to primary tumors (n = 10). A) GO terms associated with proteins upregulated in CRC metastases. B) GO terms associated with proteins downregulated in CRC metastases. GO enrichment analysis was performed using clusterProfiler enrichGO. Only GO terms supported by at least three differentially expressed proteins are shown. Dot size indicates the number of contributing proteins, and dot color indicates the adjusted p-value.

GO analysis of biological processes identified several upregulated proteins in the CRC metastases associated with negative regulation of wound healing processes (Fig. 8). This was also suggested by the downregulation of transcripts involved in wound healing seen in the RNA expression data (Supplementary Material S14). Interestingly, proteins associated with angiogenesis were differentially regulated in the CRC metastases (Fig. 8). No GO terms were significantly associated with proteins downregulated in CRC metastases.

**Fig. 8:**
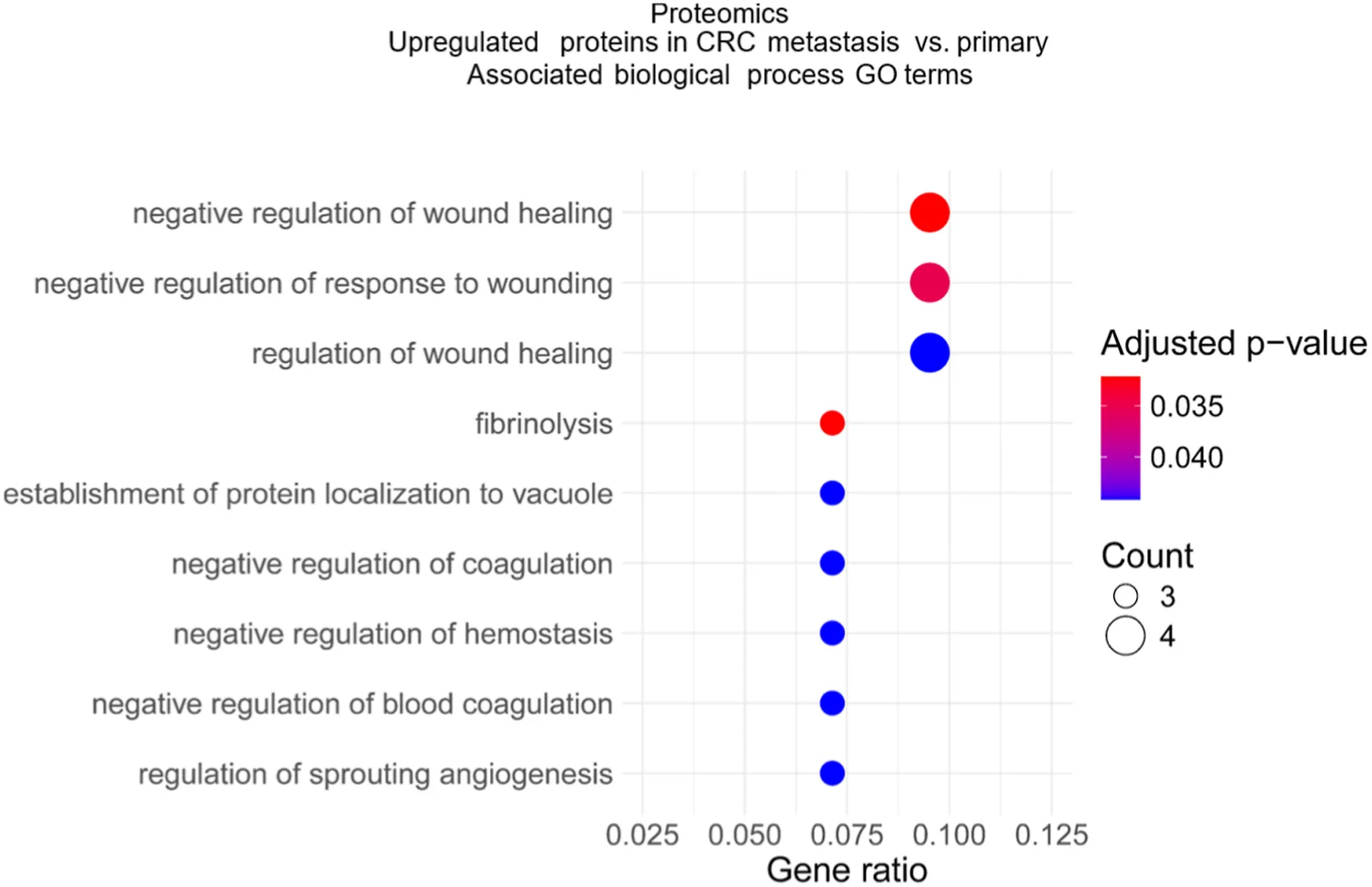
Biological process gene ontology (GO) terms associated with differentially expressed proteins in CRC metastases (n = 3) relative to primary tumors (n = 10). GO terms associated with proteins upregulated in CRC metastases. GO enrichment analysis was performed using clusterProfiler enrichGO. Only GO terms supported by at least three differentially expressed proteins are shown. Dot size indicates the number of contributing proteins, and dot color indicates the adjusted p-value.

Molecular function GO enrichment analysis of PDAC metastases compared with primary tumors identified eight upregulated proteins in the PDAC metastases related to actin filament binding, which is known to affect carcinogenesis [40] (Fig. 9A). The downregulated proteins in PDAC metastases were associated with the structure of the ribosome and cytoskeleton, GDP binding and single-stranded DNA-binding, and various enzymatic functions (Fig. 9B).

**Fig. 9:**
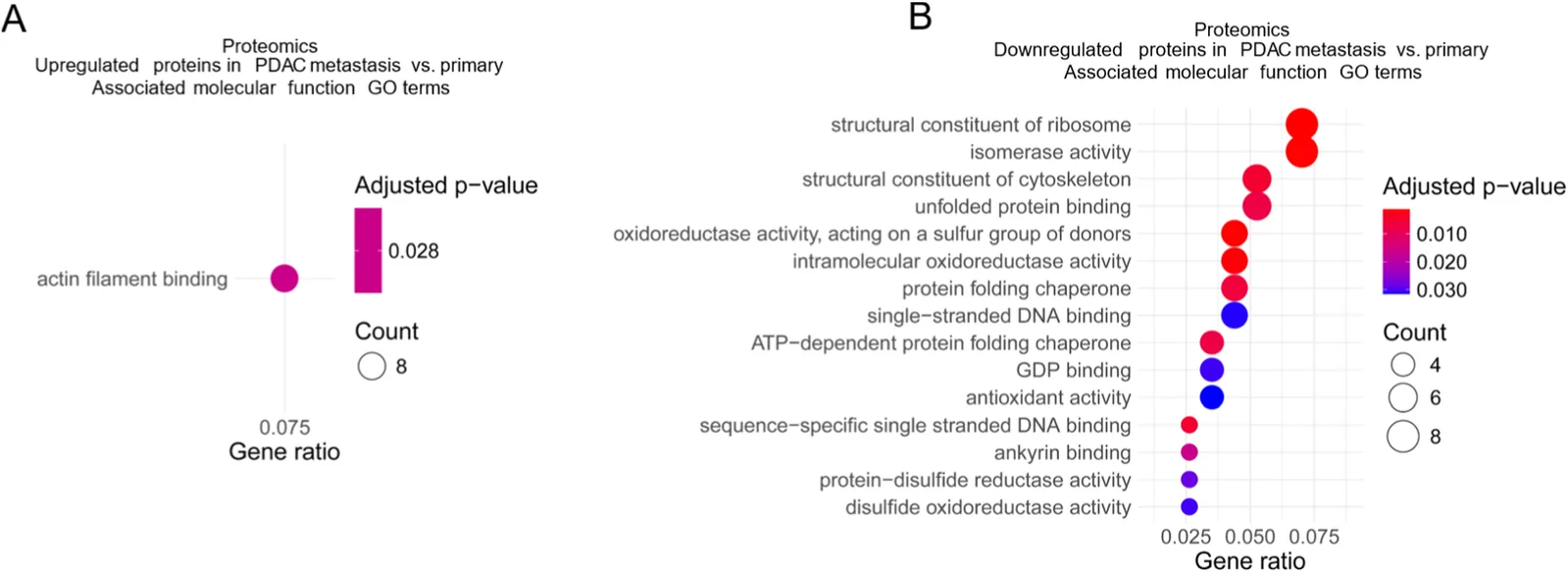
Molecular function gene ontology (GO) terms associated with differentially expressed proteins in PDAC metastases (n = 4) relative to primary tumors (n = 6). A) GO terms associated with proteins upregulated in PDAC metastases. B) GO terms associated with proteins downregulated in PDAC metastases. GO enrichment analysis was performed using clusterProfiler enrichGO. Only GO terms supported by at least three differentially expressed proteins are shown. Dot size indicates the number of contributing proteins, and dot color indicates the adjusted p-value.

Cellular components analysis showed that PDAC metastases had upregulated proteins associated with contractile fiber, myofibril, adherens junctions, and the actin filaments (Fig. 10A), while several downregulated proteins were associated with ribosomal components (Fig. 10B), as also observed for CRC metastases.

**Fig. 10:**
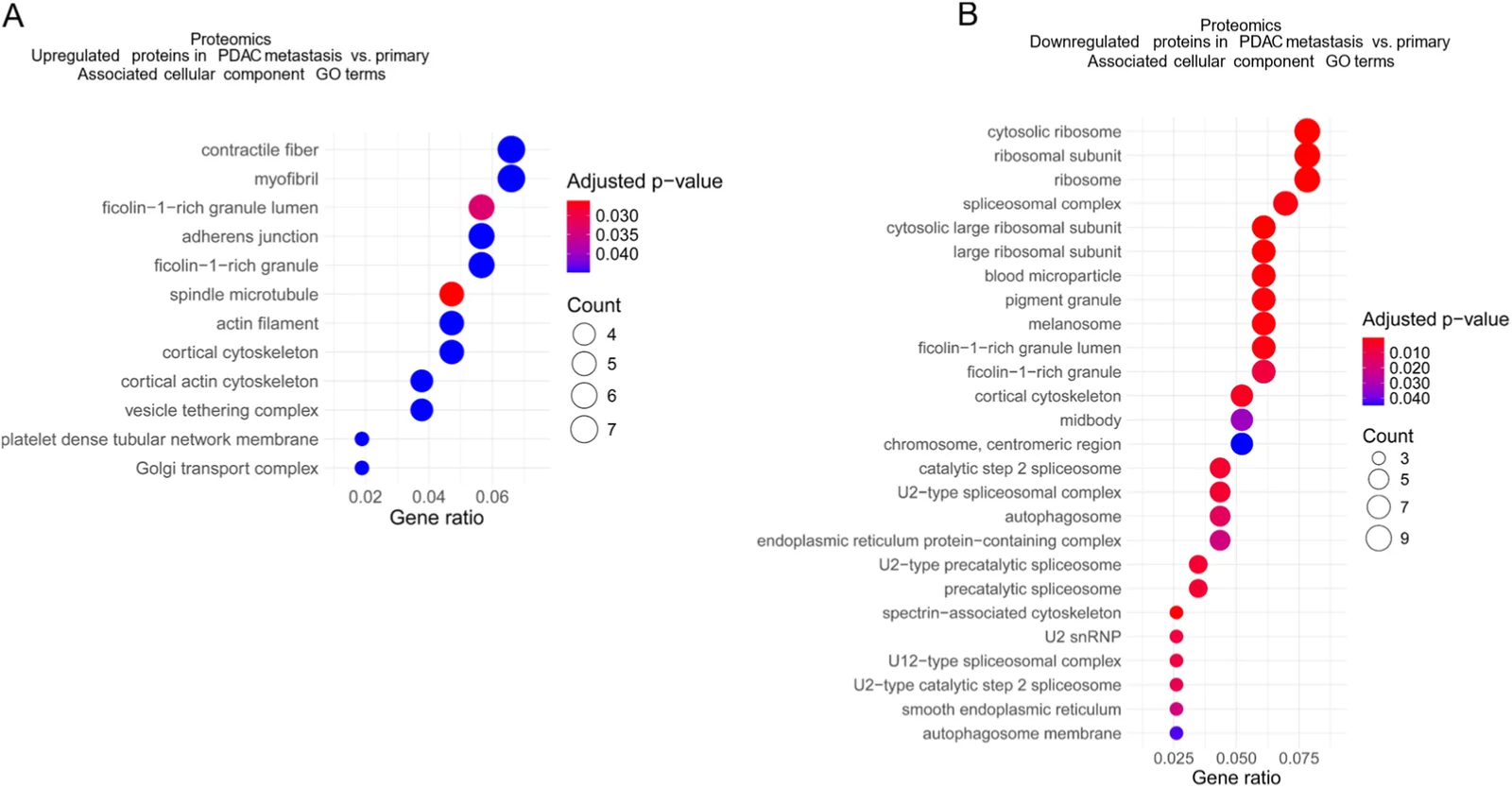
Cellular component gene ontology (GO) terms associated with differentially expressed proteins in PDAC metastases (n = 4) relative to primary tumors (n = 6). A) GO terms associated with proteins upregulated in PDAC metastases. B) GO terms associated with proteins downregulated in PDAC metastases. GO enrichment analysis was performed using clusterProfiler enrichGO. Only GO terms supported by at least three differentially expressed proteins are shown. Dot size indicates the number of contributing proteins, and dot color indicates the adjusted p-value.

GO analysis of biological processes revealed downregulation of several proteins in PDAC metastases associated with translation, regulation of actin filaments (polymerization/depolymerization), various metabolic processes, and phosphorylation (Fig. 11). No GO terms were significantly associated with proteins upregulated in PDAC metastases compared with primary tumors.

**Fig. 11:**
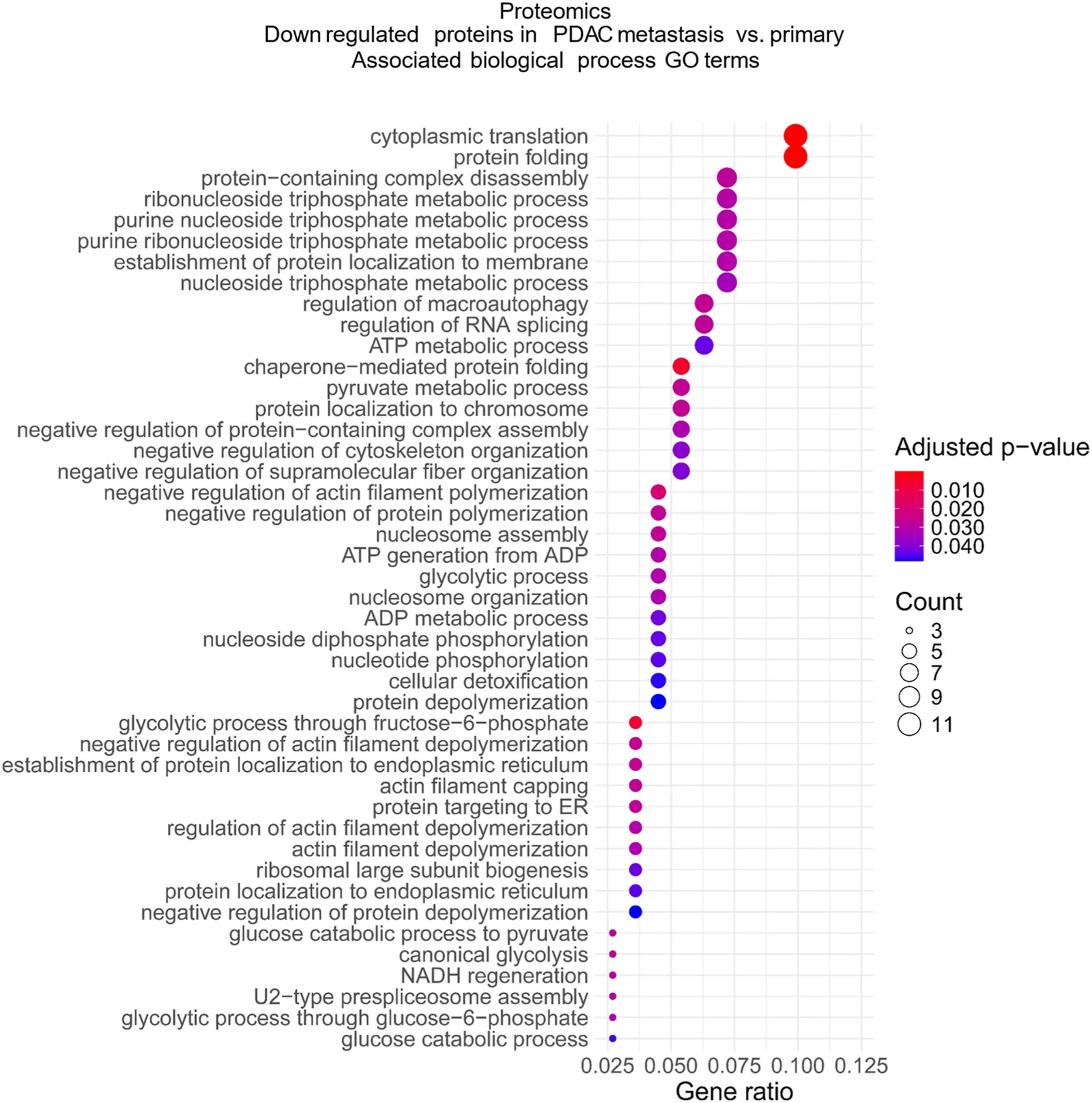
Biological process gene ontology (GO) terms associated with differentially expressed proteins in PDAC metastases (n = 4) relative to primary tumors (n = 6). GO terms associated with proteins downregulated in PDAC metastases. GO enrichment analysis was performed using clusterProfiler enrichGO. Only GO terms supported by at least three differentially expressed proteins are shown. Dot size indicates the number of contributing proteins, and dot color indicates the adjusted p-value.

### 3.5 Immune cell deconvolution with CIBERSORTx

Next, we performed unsupervised clustering of the inferred immune cell fractions (Fig. 12 and Supplementary Material S15).

**Fig. 12:**
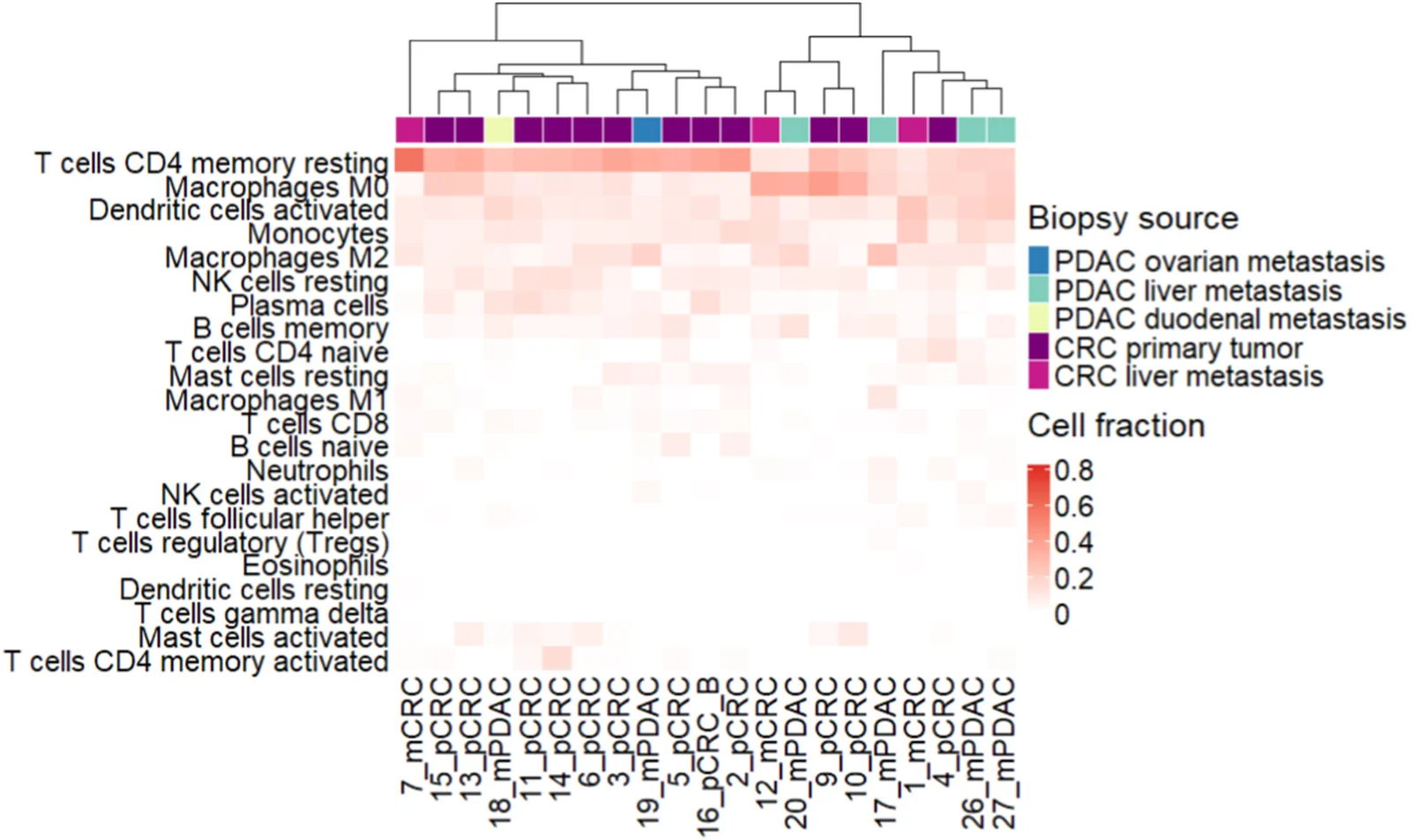
Unsupervised clustering of immune cell fractions. Inferred from RNA sequencing data, annotated with biopsy source, including sample 7_mCRC, which is from a previously treated patient. Inferred immune cell fractions were estimated from RNA sequencing data only, using the LM22 signature matrix in CIBERSORTx. Samples include CRC primary tumors (n = 12), CRC metastases (n = 3), and PDAC metastases (n = 6). No PDAC primary tumors were available for RNA sequencing.

Only samples that were RNA sequenced were immunologically characterized using CIBERSORTx and LM22. Immune cell deconvolution yielded two primary clusters, excluding the sample from the RNA sequencing in which the patient had already undergone treatment (sample 7_mCRC, Fig. 12), wherein most of the PDAC biopsies and CRC biopsies clustered together. The left cluster, consisting of CRC primary tumors and some CRC and PDAC metastases, had a majority of CD4 T memory resting cells (Fig. 12). M0 macrophages were also present in both CRC primary tumors and CRC and PDAC liver metastases.

When annotating the cell fraction heatmap with overall survival (Fig. 13), no clear relationship was observed between clinical outcomes and immune cell populations. In the CRC heatmap (Fig. 13A), two main clusters emerged, which could not be readily explained by biopsy source or clinical outcome. These clusters were primarily driven by the differences in memory resting CD4 T cells. Most CRC biopsies showed a high fraction of memory resting CD4 T cells (Fig. 13A), with the highest levels observed in the sample from a previously treated patient (sample 7_mCRC, Fig. 13A).

**Fig. 13:**
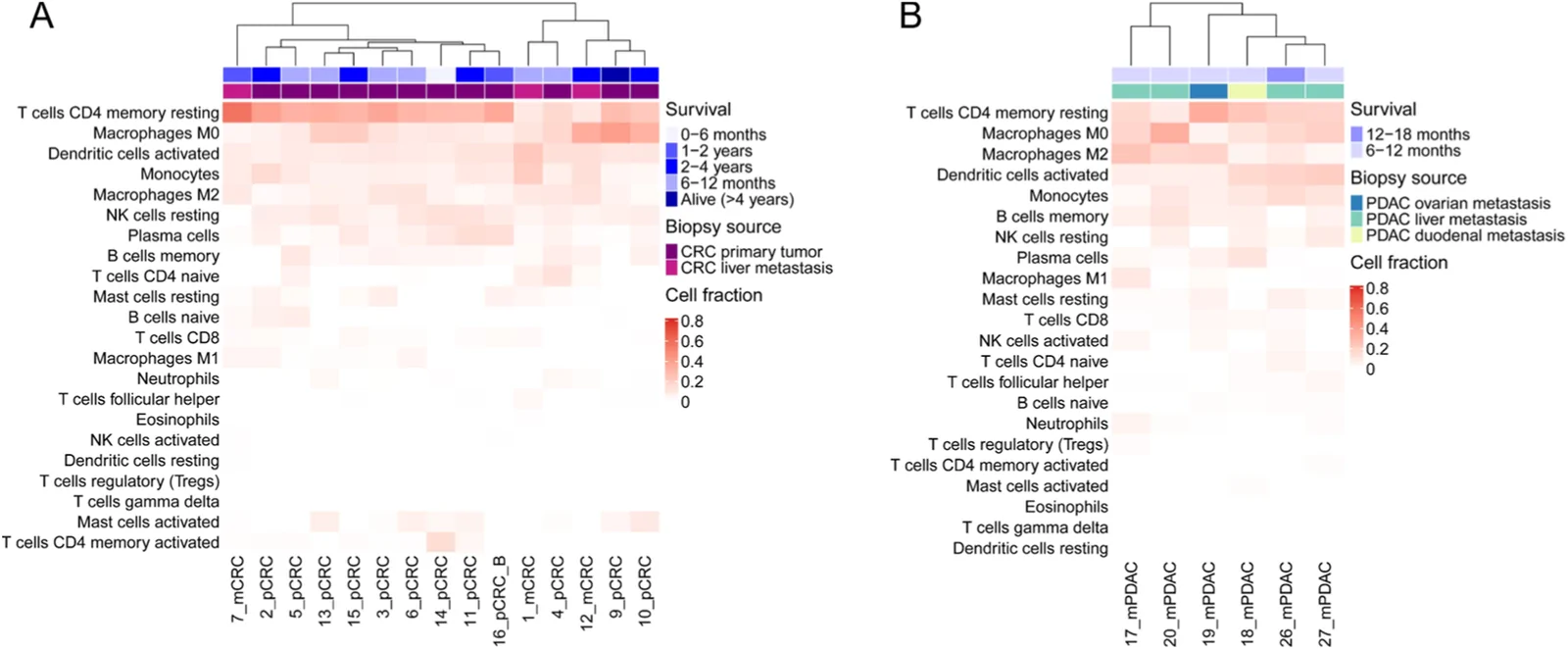
Immune cell fractions annotated with patient survival. A) Heatmap of immune cell fractions in CRC biopsies inferred from RNA sequencing data, including CRC primary tumors (n = 12) and CRC metastases (n = 3). Sample 7_mCRC is from a previously treated patient. B) Immune cell fractions in PDAC metastasis biopsies (n = 6) inferred from RNA sequencing data. Immune cell fractions were inferred using CIBERSORTx with the LM22 signature matrix.

## 4 Discussion

This study demonstrates the feasibility of performing RNA sequencing and proteomic profiling on small FFPE samples from primary tumors and metastases of CRC and PDAC. Exploratory bioinformatic analyses demonstrated differences in RNA and protein expression between metastases and primary tumors. Immune cell deconvolution indicated distinct immune cell composition between CRC primary tumors and metastases and furthermore demonstrated interpatient immune composition variability.

### 4.1 Concordance with TCGA

To contextualize our findings, we compared them with publicly available TCGA datasets, which provide large-scale transcriptomic and proteomic profiles. Overall, the mean RNA-to-protein correlation observed in our cohort was similar to that in TCGA, suggesting comparable transcript-to-protein concordance across datasets. However, in TCGA, the strongest correlations were observed among the most highly expressed transcripts and proteins. The same trend was not observed in our LC-MS-based dataset. This could reflect methodological differences, as the TCGA proteomic data were generated using RPPAs, which may preferentially capture high-abundance proteins and thus inflate correlation estimates within this subset. In contrast, LC-MS-based proteomics provides discovery-based proteomic profiling, potentially explaining the more uniform correlation distribution across transcript and protein expression levels.

In MDS analysis of TCGA COAD transcriptomic data, metastases did not form a fully distinct cluster. Although metastases tended to group together, they were embedded in a broader cluster that also contained many primary tumors, indicating large overlap in RNA expression between primary tumors and metastases. This supports the notion that COAD metastases retain much of the transcriptomic profile of their primary tumor, consistent with our preliminary observations in CRC.

Differential expression analysis of COAD samples from TCGA revealed a substantially larger number of differentially expressed transcripts between metastases and primary tumors compared with our CRC cohort. While no individual transcripts overlapped at the level of statistical significance, genome-wide FC comparisons showed moderate concordance (Spearman's ρ ≈ 0.35), indicating that similar directional changes are present but may not reach significance in smaller datasets. This discrepancy is likely driven by the much larger sample size in TCGA, which provides greater statistical power. Furthermore, relatively stringent thresholds combined with the very limited number of CRC metastasis samples likely increased the risk of false negatives. Thus, our reanalysis of the TCGA data should be interpreted as contextualization rather than direct validation of patterns observed in our datasets.

It should be noted that the TCGA cohort used for comparison comprised COAD rather than the broader CRC category, which also includes rectal tumors. While colon and rectal cancers share many molecular features and are often analyzed jointly, differences in tumor biology and treatment exposure may introduce some heterogeneity that is not captured in the TCGA COAD dataset.

### 4.2 Comparing RNA and proteomic expression in CRC and PDAC

There were clear differences in total RNA and protein expression between the two cancer types, and they were more prevalent in the protein expression. Our transcript-to-protein correlation analysis demonstrated modest concordance between RNA and protein expression. One possible explanation for this is that protein abundance is only partially determined by transcript levels and may be shaped by post-transcriptional and post-translational regulatory mechanisms, thereby providing complementary biological information. Studies comparing RNA sequencing to proteomic analysis have previously shown that the two techniques generate complementary data, where the proteomic results capture post-transcriptional changes that are not readily apparent through RNA sequencing [41], [42]. Furthermore, in an analysis of previous RNA sequencing and proteomics performed by the Clinical Proteomic Tumor Analysis Consortium (CPTAC), unsupervised clustering differed between RNA- and protein-based profiles of identical tumor samples, and the authors highlight the importance of proteomic analyses for tumor characterization [43]. Our findings support this observation, as the more distinct clustering of cancer types in the proteomic data suggests that protein composition captures tumor-specific variation more directly than RNA composition. However, it should be noted that proteomic approaches generally have lower sensitivity for low-abundance proteins, including many regulatory factors and transcription factors, which may limit their ability to detect certain biologically relevant signals [44], [45].

In unsupervised clustering of total RNA composition, CRC liver metastases clustered with the primary CRC tumors, not with PDAC liver metastases, indicating that the RNA expression of a CRC metastasis is more dependent on the primary tumor than the microenvironment of the liver. However, in the unsupervised clustering of proteomics data, all CRC liver metastases clustered together, along with two PDAC liver metastases and one PDAC duodenal metastasis. Only one PDAC liver metastasis clustered with the PDAC primary tumors. Recently, Li et al. found through integrated omics of metastatic CRC that metastases exhibited high similarities with primary tumors at the genetic, but not at the proteomic level [46]. This was also reflected in our analysis of differentially expressed transcripts in CRC metastases versus primary tumors, where the number of differentially regulated transcripts was much lower than the number of differentially expressed proteins. This is likely due to post-transcriptional changes that allow for site-specific modifications of the tumor composition. In a molecular comparison of primary tumors and metastases of PDAC, Brar et al. found no statistically significant differences at the gene or pathway level, although they did report elevated expression of two genes in pancreatic metastases, ERCC1 and TOP1 [47]. While we did not find elevated expressions of ERCC1 nor TOP1 in our analysis, they are both involved in DNA repair processes and are associated with some of the same GO terms that we found in our metastasis-to-primary tumor comparison, for example related to DNA binding. This highlights that the DNA repair and DNA binding processes, which are key mechanisms in many cancer treatment strategies [48], [49] may be altered in metastases compared with primary tumors. DNA repair and DNA damage response pathways are also relevant targets for overcoming chemotherapy resistance [50], [51]. Zhang et al. recently performed single cell transcriptomic analysis on biopsies from four PDAC patients with metastases to the liver [52], and found that the metastases exhibited different RNA expression patterns compared with their primary tumors, as we also see in our unsupervised clustering of total RNA data. They also discovered an immunosuppressive environment in PDAC metastases. In our analysis, the PDAC liver metastasis that did not cluster with the rest of the PDAC liver metastases could possibly be part of a different transcriptional trajectory as described by Zhang et al. [52]. In one case, we performed proteomic analysis on both the PDAC primary tumor and ovarian metastasis from the same patient. The total protein expression of this metastasis more closely resembled that of CRC primary tumors than the patient's own PDAC primary tumor. This observation is supported by Khaliq et al. who showed through spatially resolved transcriptomics that PDAC liver metastases are distinctly different from their primary sites, and suggest that the metastases adapt to their local environment for progression [53].

One CRC liver metastasis had a notably different RNA expression to the other liver metastases, and a more similar RNA expression to the PDAC duodenal metastasis. This patient had already undergone radiation- and chemotherapy and liver- and rectal surgery before the analyzed biopsy was taken. Radiation is known to induce changes in gene expression, including miRNA-expression, mRNA expression and lncRNA expression, all of which will contribute to the total RNA expression analyzed in our study [54], [55]. Furthermore, in a recent single-cell RNA sequencing study of PDAC, chemotherapy was shown to strongly alter the tumor microenvironment and immune cell population, selecting for more aggressive basal-like tumor cells and promoting chemotherapy resistance [56]. To our knowledge, no RNA sequencing studies have directly demonstrated transcriptional and immunological changes after chemotherapy in CRC. However, given the known alterations of RNA composition by chemotherapy and radiation, we believe that the divergent RNA composition of the sample in our study is because it is not treatment naïve.

Our findings solidify the value of proteomic analysis as a valuable complement to transcriptomic approaches. As emerging cancer therapies increasingly target specific cellular pathways, understanding protein abundance and post-translational modifications is essential for developing patient-specific treatment strategies. Proteomic analysis captures variations in tumor biology and the tumor microenvironment that may not be reflected at the transcriptomic level, offering deeper insight into the mechanisms underlying metastatic CRC and PDAC. Expanding proteomic analysis to larger cohorts could provide further insight into the metastasis process and characteristics.

Next, we performed GO analysis which revealed that a range of up- or downregulated proteins in metastases were associated with cellular components, molecular functions and biological processes relevant for metastasis. Proteins upregulated in PDAC metastases were related to actin filament and actin filament binding. Cytoskeleton constituents were downregulated, which may indicate that microtubules and intermediate filaments are differently expressed compared with actin filaments. Actin and actin binding proteins have been shown to play a large role in carcinogenesis, including migration and invasion of cancer cells [40], [57], [58]. While our proteomic data highlight pathways that could, in principle, support enhanced migratory behavior in PDAC metastases, prior functional studies have demonstrated that PDAC primary tumor cells display greater migratory activity than their metastatic counterparts [59]. Nevertheless, the regulation of actin binding proteins remains highly relevant for metastasis, especially for invasive behavior. For example, actin bundling proteins, a subgroup of actin binding proteins, such as fascin and α-actinin-4 have been shown to play a role in extracellular matrix degradation and tumor cell invasion [60]. More broadly, actin binding proteins have been suggested as potential targets for cancer therapy [40], [61]. In PDAC, where metastasis is a major indicator for poor prognosis and short survival [62], further study of the role of actin binding proteins might be one approach to better understand and treat this disease.

Proteins associated with ribosomes were downregulated in both CRC and PDAC metastases compared with the primary tumors. This could indicate less protein synthesis in the metastases, although further validation is needed to support this interpretation. The dysregulation of protein synthesis is known to play an important role in human cancer [63], [64]. Ebright et al. have previously found that overexpression of *RPL15*, a gene encoding for a component of the large ribosomal subunit, increased metastatic growth in a human breast cancer model [65].

Angiogenesis is essential for the development and growth of tumors [66]. Proteomic analysis of CRC metastases and primary tumors revealed an upregulation of sprouting angiogenesis, along with upregulation of the negative regulation of sprouting angiogenesis, possibly suggesting increased angiogenic activity in CRC metastases. This could be due to the well-vascularized environment of the liver, however, the GO analysis did not identify changes in angiogenic activity in PDAC liver metastases. The difference may reflect variations in the availability or regulation of angiogenic factors between CRC and PDAC. In metastatic CRC, angiogenesis is a validated therapeutic target where anti-VEGF agents are standard care [9], whereas clinical trials of anti-angiogenic therapies in PDAC have showed little to no improvement of overall survival [67], [68]. This limited efficacy has been attributed not only to impaired delivery of therapeutic antibodies in PDAC due to its vessel biology and tumor microenvironment [67], [69], but also to the tumor's reliance on alternative angiogenic pathways [70], [71]. Our findings support this latter explanation, indicating that even when metastasizing to the same organ, CRC and PDAC tumors may exhibit distinct expression profiles of angiogenesis-related proteins.

Interestingly, G-protein activity was upregulated in CRC metastases compared with primary tumors. The role and mechanisms of G-proteins in cancer development is not fully understood, however, they are known to play several roles in regulating tumor growth and metastasis [72]. Shen et al. report that G-protein signaling protein family modulators were elevated in CRC, and high levels correlated with worse prognosis [73].

ATP and ADP are known immunological agents that influence the tumor microenvironment, with ATP acting as a pro-inflammatory signal [74], [75], while ADP is typically found in cold tumors [76], [77], [78]. Extracellular ATP activates immune cells via P2 receptors, promoting inflammasome activation, dendritic cell maturation, and T cell priming, features commonly associated with immunogenically hot tumors like CRC. In contrast, in cold tumors such as PDAC, ATP is rapidly broken down by ectoenzymes like CD39 and CD73 into ADP and ultimately adenosine, which suppress immune responses by inhibiting T cells and promoting regulatory immune cells, maintaining the non-inflamed state of cold tumors. Targeting this pathway, such as by inhibiting CD39 or CD73, is a suggested strategy to convert cold tumors into hot tumors [76], [78], [79]. Interestingly, we found several proteins with lower expression in PDAC metastases that were associated with the ATP metabolic process, ATP generation from ADP, and the ADP metabolic process. While we did not observe evidence of altered expression of CD39 or CD73 in the PDAC metastases analyzed based on examination of respectively *ENTPD1* and *NT5E* in the differential expression analysis of our proteomic data, our results may suggest differences in ATP and ADP metabolism between PDAC metastases and primary tumors. Whether these differences reflect immunological divergence between PDAC metastases and primary tumors remains speculative without evidence of altered CD39/CD73 expression or direct ATPase activity measurements. Nevertheless, this exploratory interpretation is consistent with Steele et al., who, using single-cell transcriptomics, recently demonstrated that PDAC metastases undergo metabolic reprogramming in ATP-related pathways and display an altered immune landscape compared with primary tumors [80].

These exploratory findings suggest two candidate hypotheses for future validation. First, actin-binding and cytoskeletal proteins identified in PDAC metastases may reflect metastatic adaptation or invasive behavior and should be assessed in larger PDAC cohorts. Second, angiogenesis- and G-protein-related proteins identified in CRC metastases may be associated with CRC liver metastasis biology and should be evaluated for associations with treatment response or clinical outcome. These hypotheses require validation in independent cohorts, including assessment of whether the observed protein-level differences are supported by matched transcriptomic data, before any prognostic or therapeutic relevance can be inferred.

### 4.3 Inferred immune cell fractions

Importantly, individual samples in the present cohort were not classified as immunologically “hot” or “cold”; immune-related findings were interpreted only in terms of relative RNA-based CIBERSORTx-inferred immune cell fractions. The inferred immune cell fractions revealed that memory cells were dominating in the biopsies. Based on our exploratory analysis, resting memory CD4 T cells appear to be the most abundant immune cell in CRC primary tumors. These memory cells were also found in some CRC and PDAC metastases. Notably, the previously treated CRC metastasis (sample 7_mCRC) displayed a high fraction of memory resting CD4 T cells, which could possibly be a consequence of prior therapy, as increased infiltration of CD4 T cells has been reported after chemotherapy or chemotherapy in combination with radiotherapy in some cancers, including gastric, rectal, and pancreatic cancer [81].

Bioinformatic frameworks like CIBERSORTx enable estimation of immune cell fractions from bulk tissue data and are used to identify infiltration of subgroups of leukocytes. In a pan-cancer analysis of 14 non-brain cancers, resting memory CD4 T cells ranked second among leukocytes most strongly associated with improved survival, identifying them as a robust prognostic biomarker, whereas memory B cells correlated with poorer outcomes [23]. In PDAC specifically, a comparison of inferred cell fractions from tumor and adjacent normal tissue revealed a smaller fraction of memory B cells but an increase in resting memory CD4 T cells in tumor tissue [82]. Consistent with the described role of resting memory CD4 T cells and memory B cells, our cohort of PDAC patients exhibited an overall short survival.

Zhang et al. performed single-cell RNA sequencing on metastatic PDAC and used CIBERSORTx to infer immune cell compositions [52]. The authors reported that the heterogeneity of immune cell composition was larger across tissue types (normal tissue, primary tumor and liver metastases) than across patients within one tissue type. Although we could not perform RNA sequencing on any PDAC primary tumors, our GO analysis suggests that this could be the case for our cohort too, as PDAC metastases differed from primary tumors with respect to GO terms associated with the known immunological agents ATP and ADP.

Imputation of immune cell composition has also revealed that immune cells play an essential role in CRC prognosis. Wu et al. have recently reported that, among others, memory B cells and macrophages in the M0 and M1 states were statistically correlated with CRC-related survival [20]. In agreement with what we found for PDAC, resting memory CD4 T cells were found to be highly infiltrated into colon cancer with good prognosis, whereas activated memory T cells were associated with poor prognosis [19]. Interestingly, in our CRC cohort, a patient with short survival (below 6 months) had the highest fraction of activated memory CD4 T cells. Tanaka et al. recently described 6 proteogenomic subtypes of primary tumors and metastases in CRC, characterized partly by immune signature. Interestingly, they reported that T-cell metrics are more robust as prognostic biomarkers in CRC metastases than primary tumors [83]. This underscores the importance of considering both the metastasis site and biopsy source when applying transcriptomic and proteomic profiling, as well as for the subsequent deconvolution of immune cell populations.

### 4.4 Limitations

This study is limited by the small number of patients as well as the limited amount of tissue available from PDAC primary tumors, which prevented RNA sequencing of these samples, making transcriptomic comparison of PDAC metastases versus primary tumors infeasible. Because RNA was derived from archival FFPE material and showed variable DV200 values and low RNA integrity (median RIN = 2.3), RNA degradation may have reduced sensitivity for detecting low-abundance transcripts. Transcriptomic findings, particularly for lowly expressed genes, should therefore be interpreted cautiously. Furthermore, the small group sizes, particularly for RNA-sequenced CRC liver metastases (n = 3) together with the rather stringent cutoffs chosen for the DE analysis, increase the risk of false negatives, and the transcriptomic comparison of CRC liver metastases versus primary tumors should be viewed as preliminary. This is particularly relevant in the context of FFPE-related RNA degradation, which may have further reduced sensitivity for detecting low-abundance transcripts and contributed to the low number of identified differentially expressed transcripts (n = 5) in the comparison of CRC metastases versus primary tumors. When comparing publicly available RNA sequencing data from CRC metastases versus primary tumors, more transcripts were differentially expressed than in our analysis. The small CRC liver metastasis group size and the lack of PDAC primary tumor RNA sequencing also prevent assessment of whether the observed proteomic differences are mirrored at the transcriptomic level. Despite this constraint, important comparisons between primary tumors and metastases were achieved. Another limitation is the lack of direct comparisons between primary tumors and metastases in the same patient, with the exception of the proteomic comparison of one patient's primary PDAC tumor and ovarian metastasis. Metastases from different metastasis sites may also confound the comparison between metastases and primary tumors of the same cancer type, and findings should not be interpreted as reflecting metastatic biology alone. Inferred immune cell populations were based on RNA sequencing data, as the LM22 signature matrix used is based on transcriptomic profiles, and this study therefore does not provide insight into the immunological status of PDAC primary tumors. Additionally, our study did not directly assess tumor heterogeneity. Bulk RNA sequencing cannot capture cell-to-cell variation within tumors, potentially obscuring differences in gene expression and immune composition among subpopulations.

A key strength of our study was that all biopsies were obtained from patients enrolled in the same clinical trials, either the CRC or PDAC trial. Moreover, although metastases from different metastasis sites complicate direct metastasis-to-primary tumor comparison, they also provide some insight into the importance of metastasis site for both RNA and proteomic expressions.

## 5 Conclusions

This exploratory study demonstrates the feasibility of RNA sequencing and proteomic profiling from limited FFPE diagnostic biopsies from patients with CRC and PDAC. In CRC, RNA sequencing and proteomic analysis revealed differences between primary tumors and metastases, while PDAC metastasis-to-primary tumor comparisons were limited to proteomic data because primary PDAC biopsies were not available for RNA sequencing. Across analyses, proteomic profiles showed greater divergence by biopsy source than RNA profiles, supporting the complementary value of proteomic and transcriptomic profiling.

Several differentially expressed proteins mapped to processes related to extracellular matrix organization, protein synthesis, adherens junctions, cytoskeletal organization, angiogenesis, and immune-related pathways. However, given the small and unbalanced cohort, these findings should be considered hypothesis-generating and require validation in larger independent cohorts and by orthogonal methods. Future studies should assess whether selected proteomic features, including actin-binding and cytoskeletal proteins in PDAC metastases and angiogenesis- or G-protein-related proteins in CRC metastases, are associated with metastatic disease biology, treatment response, or clinical outcome. These candidate findings should be validated in larger independent cohorts, including matched transcriptomic and proteomic datasets where available, before any prognostic or therapeutic relevance can be inferred.

## Availability of data and materials

Under Norwegian law on sensitive data, raw patient-derived sequencing data are considered personal health data and cannot be submitted to public repositories without explicit participant consent and additional regulatory approvals. All processed and aggregated data necessary to support our findings are included in the manuscript and supplementary materials. Access to raw sequencing data that are not part of the main manuscript or supplementary material may be considered upon reasonable request to the corresponding author, subject approval by the Regional Committee for Medical and Health Research Ethics Central Norway (REK Central) and relevant institutional data protection authorities.

## Authors’ contributions

VN designed the study, performed data analyses, contributed to data interpretation and conceptualizing, drafting and editing the manuscript. MH contributed to the acquisition and interpretation of clinical data and assisted in editing the manuscript. EH contributed to the acquisition and interpretation of clinical data, interpretation of data and conceptualizing and editing the manuscript. ODR contributed to the interpretation of data and assisted in conceptualizing and editing the manuscript. CD designed the study and contributed to the interpretation of data and conceptualizing, drafting and editing the manuscript. RM designed the study, contributed to data analysis and interpretation of data and conceptualizing and editing the manuscript. All authors read and approved of the final manuscript.

## Ethics declaration

The authors have NOT obtained informed consent from participants or their legal representatives. All diagnostic biopsy samples analyzed in this study were obtained from patients who had previously provided informed consent to participate in two clinical studies. Diagnostic biopsies were obtained and stored prior to these studies as part of standard clinical practice. Following the guidance of The Regional Committee for Medical and Health Research Ethics Central Norway (REK Central), surviving patients were given the opportunity to reserve themselves from having their already collected samples used in this study, however, none chose to do so. For deceased patients, REK Central advised that additional consent was not required.

This study included organ or tissue donors. This study includes human biological material and consent was obtained by donors, or their next of kin or legal representatives, for use in this study and for publication of the article. The samples used in this research were not sourced from executed prisoners or prisoners of conscience.

This study was performed in compliance with relevant laws, regulatory frameworks and guidelines where the research took place. This study was approved by the Regional Committee for Medical and Health Research Ethics Central Norway (REK Central). (Approval No. 533301)

## Funding

This project is funded by the 10.13039/100018696Central Norwegian Health Authority (project number 30288) and supported by a grant from 10.13039/100019902The Joint Research Committee between St. Olavs hospital and the Faculty of Medicine and Health Sciences, NTNU (FFU) (Grant# 46055600–113 and 46055600–134) and from the post-doc grant from The Liaison Committee for Education, Research and Innovation in Central Norway (Samarbeidsorganet) (Grant# 2020/39645).

## Declaration of competing interest

The authors declare that they have no competing interests.

## List of abbreviations

CRC –: Colorectal cancer
PDAC –: Pancreatic ductal adenocarcinoma
ICIs –: Immune checkpoint inhibitors
VEGF –: Vascular endothelial growth factor
EGFR –: Epidermal growth factor receptor
MSI-high –: Microsatellite instability-high
MSS –: Microsatellite stable
FFPE –: Formalin-fixed, paraffin-embedded
GCF –: Genomic Core Facility
PROMEC: Proteomics and Modomics Experimental Core Facility
LC-MS –: Liquid-chromatography mass spectrometry
TMM –: Trimmed mean of M-values
ENSG –: Ensembl gene identifiers
MDS –: Multidimensional scaling
FC –: Fold change
HGNC –: HUGO gene nomenclature committee
GO –: Gene ontology
