# Supplementary material for: RNA sequencing and proteomic analysis of diagnostic biopsies from colorectal and pancreatic cancer
Source: Biochem Biophys Rep. 2026 Nov 2;48:102846. doi: 10.1016/j.bbrep.2026.102846 (PMC13745044; doi:10.1016/j.bbrep.2026.102846)
Supplement: Supplementary file 1 [file mmc2.docx]

# Supplementary Material

### S2: RNA isolation

RNA isolation was performed by Biobank1 using the RNeasy FFPE kit (QIAgen^TM^, Hilden Germany), employing a half-automated isolation of totalRNA from FFPE on the QIAcube^TM^ according to the manufacturer’s instructions. The sections were first treated with deparaffinization solution and lysated. Subsequently, they were DNase-treated, and the lysate was washed in the QIAcube^TM^, resulting in a washed RNA eluate. The Nanodrop and Qubit were used to measure the concentration and purity of the eluate. Agilent 2100 Bioanalyzer (Agilent Technologies) was used to attain a RIN-score and assess the degree of RNA fragmentation.

### S3: RNA sequencing

RNA sequencing and library preparation was performed by the Genomic Core Facility (GCF).

RNA sequencing libraries were prepared using Illumina Stranded Total RNA Prep, ligation with Ribo-Zero kit (Illumina, San Diego, CA, USA) according to the manufacturer's instructions.

In brief, 100 ng total RNA was used as starting material. Abundant rRNA was depleted using DNA probes to bind rRNA targets, which were enzymatically digested to produce total RNA free from abundant transcripts. To recover and purify the rRNA-depleted RNA, RNA-specific magnetic beads were used.

Fragmentation of the purified total RNA, and annealing of random hexamers, was performed at 94°C for 2 minutes, following first strand cDNA synthesis using reverse transcriptase and the First Strand Synthesis Mix (FSA) included in the kit. The FSA includes Actinomycin D, which allows RNA-dependent synthesis and improves strand specificity. During second strand cDNA, the RNA template was removed, and a replacement strand was synthesized to generate blunt-ended, double-stranded cDNA. In place of deoxythymidine triphosphate (dTTP), deoxyuridine triphosphate (dUTP) was incorporated to quench the second strand during amplification and achieve strand specificity.

Following adenylation, where an adenine (A) nucleotide was added to the 3’ ends of the blunt fragments, pre-index anchors were ligated to the ends of the double-stranded cDNA fragments to prepare them for dual indexing. 15 cycles of PCR were used to amplify anchor-ligated DNA fragments and indexes (Illumina® RNA UD Indexes Set D, Ligation, Illumina, San Diego, CA, USA) and primer sequences for cluster generation were added.

The libraries were purified using the Mag-Bind TotalPure NGS (Omega Biotek, Norcross, GA, USA), quantitated by qPCR using Collibri™ Library Quantification Kit (Thermo Fisher Scientific, MA, USA) and validated using High Sensitivity DNA ScreenTapes on the Agilent TapeStation 4200 (Agilent Technologies, Santa Clara, CA, USA). The size range of the DNA fragments were measured to be in the range of 200-500 bp and peaked at around 275 bp.

Prior to sequencing, the libraries were normalized and pooled before excess adapter dimers were removed from using Mag-Bind TotalPure NGS (Omega Biotek, Norcross, GA, USA). The pool was quantified (Collibri™ Library Quantification Kit, Thermo Fisher Scientific, MA, USA) and further diluted to 200 pM, clustered and sequenced on a NovaSeq6000 SP flowcell (Illumina Inc. San Diego, CA, USA) R1: 59 bp, R2: 10 bp, R3: 10 bp and R4 59 bp, according to manufacturer’s instructions. FASTQ files were created with bcl2fastq V2.20 (Illumina, Inc., San Diego, CA, USA). FASTQ files quality controlled with fastqc (v0.11.9) then filtered and trimmed by fastp (v0.20.0). Trimmed sequences were aligned to the genome reference using STAR (v2.7.3) and quality metrics were extracted with picard CollectRNASeqMetrics (v2.21.5). Transcript counts were generated using quasi alignment (Salmon v1.3.0) to the GRCh38 transcriptome reference sequences. Transcript counts were imported into the R statistical software and aggregated to gene counts using the tximport (v1.14.0) bioconductor package for downstream statistical analysis.

### S4: Proteomic analysis

Liquid chromatography mass spectrometry-based identification and quantification of proteins was performed by the Proteomics and Modomics Experimental Core Facility (PROMEC). Samples were deparaffinized with xylene and further washed with 70% ethanol and water. The deparaffinized samples were added 100µl 1% SDC, 100mM Tris-HCl pH-8.5, 10mM TCEP, 40mM CAA, before further sonication for 20 cycles (30 sec ON/30 sec OFF) using the Bioruptor pico sonicator. Samples were heated for 2hr at 80°C. Debris were spun down (16000 g, 10 min) and sample was added 100 µl 0.1M Ammonium bicarbonate, 500 ng trypsin and digested overnight at 37°C. Peptides were desalted using C18 spin columns, dried in a speedvac centrifuge and resuspended in 20ul 0.1% Formic acid before MS analysis. LC-MS/MS were performed on a timsTOF Pro (Bruker Daltonics) connected to a nanoElute (Bruker Daltonics) HPLC. Peptide separation was done using a Pepsep 25 (150um*25cm) column with running buffers A (0.1% formic acid) and B (0.1% formic acid in acetonitrile) with a gradient from 2% B to 40%B for 100min. The timsTof instrument was operated in the DDA PASEF mode with 10 PASEF scans per acquisition cycle and accumulation and ramp times of 100 ms each. The ‘target value’ was set to 20,000 and dynamic exclusion was activated and set to 0.4 min. The quadrupole isolation width was set to 2 Th for m/z < 700 and 3 Th for m/z > 800.

### S5: Comparison of RNA and protein expression levels

S5: RNA and protein expression comparison. A) Spearman correlation coefficients between RNA and protein expression levels of matched genes from RNA sequencing and proteomic data. B) The twenty genes with the strongest positive and twenty with the strongest negative correlations. C) The absolute Spearman correlation coefficients for groups binned by log-transformed protein expression levels and log-transformed RNA expression levels.

### S6: Comparison of RNA and protein expression levels in TCGA datasets

S6: RNA and protein expression comparison of transcripts and proteins in TCGA datasets. A) Spearman correlation coefficients between RNA and protein expression levels of matched genes from RNA sequencing and proteomic data. B) The twenty genes with the strongest positive and twenty with the strongest negative correlations. C) The absolute Spearman correlation coefficients for groups binned by log-transformed protein expression levels and log-transformed RNA expression levels.

### S7: Total RNA and protein correlation excluding 7_mCRC

S7: Overall RNA expression excluding 7_mCRC. Overall expression of RNA in biopsies shown as a heatmap of the Pearson correlation coefficient (r) in sample-to-sample comparisons, with previously treated sample 7_mCRC excluded.

### S8: Total RNA and protein correlation annotated with overall survival

S8: Transcript and protein correlations with survival. A) Overall expression of RNA in CRC biopsies shown as a heatmap of the Pearson correlation coefficient (r) in sample-to-sample comparisons. B) Overall expression of protein in CRC biopsies shown as a heatmap of the Pearson correlation coefficient (r) in sample-to-sample comparisons. C) Overall expression of RNA in PDAC biopsies shown as a heatmap of the Pearson correlation coefficient (r) in sample-to-sample comparisons. D) Overall expression of protein in PDAC biopsies shown as a heatmap of the Pearson correlation coefficient (r) in sample-to-sample comparisons.

### S9: Multidimensional scaling analysis excluding 7_mCRC

S9: Two-dimensional (2D) multidimensional scaling (MDS) projection of overall RNA expression (dim 1 = dimension 1, dim 2 = dimension 2), with treated sample 7_mCRC excluded.

### S10: Multidimensional scaling analysis TCGA

S10: Two-dimensional (2D) multidimensional scaling (MDS) projection of overall RNA expression (dim 1 = dimension 1, dim 2 = dimension 2).

### S12: Differentially expressed transcripts in metastases in COAD RNA sequencing

S12: Differential expression analysis of COAD metastases compared to primary tumors. Volcano plot showing differentially expressed transcripts (the most differentially expressed mapped to gene names) in metastases relative to primary tumors in TCGA COAD dataset. The blue and red dots represent proteins that are respectively downregulated or upregulated in metastases compared to the primary tumors.

### S15: Inferred immune cell fractions

S8: All immune cell fractions inferred from RNAseq and proteomic data, annotated by biopsy source.
